# Snorkel‐tag based affinity chromatography for recombinant extracellular vesicle purification

**DOI:** 10.1002/jev2.12523

**Published:** 2024-10-14

**Authors:** Madhusudhan Reddy Bobbili, André Görgens, Yan Yan, Stefan Vogt, Dhanu Gupta, Giulia Corso, Samir Barbaria, Carolina Patrioli, Sylvia Weilner, Marianne Pultar, Jaroslaw Jacak, Matthias Hackl, Markus Schosserer, Regina Grillari, Jørgen Kjems, Samir EL Andaloussi, Johannes Grillari

**Affiliations:** ^1^ Institute of Molecular Biotechnology, Department of Biotechnology BOKU University Vienna Austria; ^2^ Ludwig Boltzmann Institute for Traumatology The Research Center in Cooperation with AUVA Vienna Austria; ^3^ Austrian Cluster for Tissue Regeneration; ^4^ Department of Laboratory Medicine, Division of Biomolecular and Cellular Medicine Karolinska Institutet Stockholm Sweden; ^5^ Department of Cellular Therapy and Allogeneic Stem Cell Transplantation (CAST) Karolinska University Hospital Huddinge and Karolinska Comprehensive Cancer Center Stockholm Sweden; ^6^ Institute for Transfusion Medicine, University Hospital Essen University of Duisburg‐Essen Essen Germany; ^7^ Department of Molecular Biology and Genetics, Centre for Cellular Signal Patterns (CellPat), Interdisciplinary Nanoscience Centre (iNANO) Aarhus University Aarhus C Denmark; ^8^ Omiics ApS Aarhus N Denmark; ^9^ Evercyte GmbH Vienna Austria; ^10^ TAmiRNA TAmiRNA GmbH Vienna Austria; ^11^ School of Medical Engineering and Applied Social Science University of Applied Sciences Upper Austria Linz Austria; ^12^ Institute of Medical Genetics Center for Pathobiochemistry and Genetics Medical University of Vienna Vienna Austria; ^13^ Institute of Developmental and Regenerative Medicine University of Oxford, IMS‐Tetsuya Nakamura Building, Old Road Campus, Roosevelt Dr, Headington Oxford United Kingdom; ^14^ Department of Paediatrics University of Oxford, South Parks Road Oxford United Kingdom

**Keywords:** affinity chromatography, CD81, extracellular vesicles, snorkel‐tag, StEVAC, tetraspanins

## Abstract

Extracellular vesicles (EVs) are lipid nanoparticles and play an important role in cell‐cell communications, making them potential therapeutic agents and allowing to engineer for targeted drug delivery. The expanding applications of EVs in next generation medicine is still limited by existing tools for scaling standardized EV production, single EV tracing and analytics, and thus provide only a snapshot of tissue‐specific EV cargo information. Here, we present the Snorkel‐tag, for which we have genetically fused the EV surface marker protein CD81, to a series of tags with an additional transmembrane domain to be displayed on the EV surface, resembling a snorkel. This system enables the affinity purification of EVs from complex matrices in a non‐destructive form while maintaining EV characteristics in terms of surface protein profiles, associated miRNA patterns and uptake into a model cell line. Therefore, we consider the Snorkel‐tag to be a widely applicable tool in EV research, allowing for efficient preparation of EV standards and reference materials, or dissecting EVs with different surface markers when fusing to other tetraspanins in vitro or in vivo.

## 1 INTRODUCTION

Extracellular vesicles (EVs) are lipid bilayer nanoparticles secreted by all cells from prokaryotes to eukaryotes. EVs play a key role in intercellular communication, transferring signals and bioactive molecules to recipient cells or tissues and thus modulating a plethora of functions. EVs are heterogenous in nature with sizes ranging from 30 to 1000 nm and are broadly classified into two classes: exosomes that are <150 nm in diameter formed within multivesicular bodies (MVBs) and released upon fusion to plasma membrane, and the ectosomes >150 nm released by plasma membrane budding (Kalluri & Lebleu, 2020; Latifkar et al., 2019). Exosomes and ectosomes can be identified by the presence of multiple cellular components such as tumour susceptibility gene 101 protein (TSG101), Alix, syndecan–syntenin complexes and tetraspanin family (CD63, CD9 and CD81), involved in their biogenesis (Baietti et al., 2012; Larios et al., 2020; van Niel et al., 2011). While the tetraspanins CD9, CD63 and CD81 are highly enriched on the surface of all classes of EVs (Escola et al., 1998; Fordjour et al., 2022; Théry et al., 1999), more recent studies observe higher enrichment of CD63 on endosome‐derived exosomes, while plasma membrane‐derived ectosomes mostly bear CD9 (Kowal et al., 2016; Mathieu et al., 2021). CD81 appears to be highly enriched in both exosomes, and to some extent, ectosomes (Mathieu et al., 2021).

EVs that are derived from parent mesenchymal stem cells (MSC) with low immunogenicity (Zhang & Cheng, 2023) have therapeutic applications in a broad range of preclinical disease models. In acute respiratory distress syndrome (ARDS) an MSC‐EV product has even reached clinical phase III trials (https://clinicaltrials.gov; NCT05354141), Also, their unique ability to cross blood‐tissue barriers, makes it possible to engineer EVs for targeted drug‐delivery (Alvarez‐Erviti et al., 2011; Herrmann et al., 2021).

Moreover, EVs have the potential to be powerful biomarkers, due to their presence in liquid biopsies including blood, urine, cerebrospinal fluid (CSF) or synovial fluid. Thereby, their diverse cell sources, EV quantity, functional properties and cargo including nucleic acids, lipids, glycans, proteins, and metabolites that change under various physiological and pathophysiological conditions (Burbidge et al., 2020; Kumar et al., 2024; Lucotti et al., 2022; Yuana et al., 2013), relaying invaluable diagnostic and prognostic information in various diseases (Li et al., 2021; Nair et al., 2023; Ramirez‐Garrastacho et al., 2022). However, our understanding of regulatory mechanisms of EV biogenesis, cargo loading, and uptake is still limited (Hendrix et al., 2023). Several tools have been established to affinity purify EVs based on their molecular composition or surface charge. For instance, ion exchange chromatography exploits the net charge on EV surfaces to bind the EVs to chromatographic matrices, after which, the bound EVs are eluted using high salt concentrations (Chen et al., 2018; Fang et al., 2020; Heath et al., 2018; Kim et al., 2016; Saari et al., 2023; Seo et al., 2022). Affinity purification methods take advantage of specific features of the molecular composition of EVs, for example by using Tim4 to bind phosphatidyl serine (Nakai et al., 2016), lectins for heparin (Balaj et al., 2015; Barnes et al., 2022), peptides specific to EV surface proteins (Pham et al., 2021), antibodies which capture specific antigen (Kowal et al., 2016), and membrane binders including annexin V, and Shiga toxin B subunit (Lai et al., 2016). Importantly, all of these methods require a destructive elution, limiting the downstream analysis to a bulk quantification of EV compositions. In contrast, we are the first to present a tool to unambiguously affinity purify EVs in a non‐destructive manner, opening the path to single EV analysis from cell culture supernatants, from biological samples including tissues and biofluids such as serum, plasma, CSF or urine.

To harness the therapeutic potential of EVs, it is a pre‐requisite to reliably track the biodistribution of EVs *in vivo* in a quantifiable way for clinical applications. Several pre‐clinical studies have addressed the pharmacokinetics and biodistribution of EVs by directly labelling EVs with fluorescent lipophilic dyes such as DiR, DiD and PKH26/67 (Kamerkar et al., 2017; Morales‐Kastresana et al., 2017; Peinado et al., 2012). However, fluorescent lipid dyes have a longer half‐life than the EVs themselves, and are reported to form nano‐sized micelles resulting in inaccurate spatiotemporal detection of EVs (Lai et al., 2015; Pužar Dominkuš et al., 2018). Alternatively, fluorescent and bioluminescent reporters such as PalmtdTomato (Lai et al., 2015), nanoluciferase (Gupta et al., 2020), mCherry (Lázaro‐Ibáñez et al., 2021), and palmGRET (Wu et al., 2020) can be genetically fused to proteins and lipids found to be enriched in EVs. However, the use of fluorescent reporters is limited by low tissue penetration of signals and high background through tissue autofluorescence (Mittelbrunn et al., 2011; Schaub et al., 2015). Radioactive tracers such as technetium hexamethyl propylene amine oxime (^99m^Tc‐HMPAO) (Hwang et al., 2015), iodine‐131 (Hong et al., 2021), indium‐111‐oxine (^111^In) (Faruqu et al., 2019) and ^89^Zirconium deferoxamine ([^89^Zr]Zr‐DFO) (Patel et al., 2022) have also been applied to label EVs for pharmacokinetics and biodistribution analysis using single‐photon emission computed tomography (SPECT), positron emission computed tomography (PET). However, use of radioisotopes comes with limitations as it requires certified facilities and well‐trained operators, making these techniques costly and inaccessible.

Finally, there is a lack of appropriate reference materials to calibrate, normalize and develop separation methods for biofluids containing EVs. Currently available calibration materials like polystyrene beads or liposomes lack EV‐like properties (Valkonen et al., 2017; Van Der Pol et al., 2014), making them much inferior to biological EV reference materials. Nano‐biotechnological approaches have been used to develop biological references: synthetic nanovesicles with improved EV‐like characteristics, such as EV‐mimetics (Görgens et al., 2019; Lozano‐Andrés et al., 2019; Varga et al., 2018) and recombinant EVs (rEVs) which use the retroviral gag polyprotein C‐terminally fused to EGFP. However, when these rEVS are spiked into biofluids to estimate the endogenous EV recovery efficacy, this results in co‐isolation of contaminants, and requires additional modification of rEVs for validation (Geeurickx et al., 2019).

Here, we report on a versatile and broadly applicable pre‐clinical tool for generating EVs that carry a CD81 fusion protein for display of a series of tags on the EV surface. These tags enable labelling and non‐destructive purification of the associated EV via proteolytic cleavage from affinity columns. We show that the physical and biochemical characteristics of EVs do not change by recombinantly expressing the CD81‐Snorkel‐tag fusion protein. Internalization of purified EVs covalently labelled with CLIP substrates was confirmed using live‐cell confocal microscopy and flow cytometry. Moreover, EVs expressing the Snorkel‐tag was spiked into human plasma and platelet concentrate, and isolated in a non‐destructive manner. Thus, this tool provides a potential benefit for a wide range of EV applications ranging from EV tracking in vitro or in vivo, the use as reference material and the assessment of EV functionality and cargo of specific subsets of EVs that can be affinity purified.

## 2 MATERIALS AND METHODS

### 2.1 Cell culture

Cell culture experiments were performed under sterile and antibiotic free conditions. Human dermal fibroblasts (HDFs) from adult skin of one healthy donor (HDF76), and WJ‐MSC/TERT273 were provided by Evercyte GmbH. HDFs were grown in DMEM/Ham's F‐12 (1:1 mixture) (BIOCHROME, Germany) supplemented with 10% foetal calf serum (FCS) and 4 mM l‐glutamine (Sigma Aldrich GmbH St Louis, MO, USA) at 5% CO_2_ and 37°C. WJ‐MSC/TERT273 were cultivated in Mesencult with supplements (Stem cell technologies, Canada) at 5% CO_2_ and 37°C. HEK293 cells were cultivated in DMEM with Na‐pyruvate (BIOCHROME, Germany) supplemented with 10% foetal calf serum (FCS) and 4 mM l‐glutamine (Sigma Aldrich GmbH St Louis, MO, USA) at 5% CO_2_ and 37°C. Huh‐7 cells were cultivated in DMEM (ThermoFisher Scientific) supplemented with 10% foetal calf serum (FCS) (Sigma Aldrich GmbH St Louis, MO, USA) and 1 X GlutaMAX (ThermoFisher Scientific) at 5% CO_2_ and 37°C. HeLa cells were grown in RPMI 1640 (ThermoFisher Scientific) supplemented with 10% foetal calf serum (FCS) (Sigma Aldrich GmbH St Louis, MO, USA) and 1 x GlutaMAX (ThermoFisher Scientific) at 5% CO_2_ and 37°C.

### 2.2 Generation of stable cell lines

CD81‐Snorkel‐tag, the transgene was codon optimized and bought from IDT (integrated DNA technologies, USA). The sequence was cloned into the pLVX‐IRES‐Hygro vector using SpeI and NotI restriction sites to form the pLVX‐IRES‐Hygro‐CD81‐Snorkel‐tag. Sanger sequencing confirmed the correct constrct, and the CD81‐Snorkel‐tag sequence has been submitted to GenBank (accession number PQ063272). Lenti‐X™ 293T cells (TaKaRa; Cat.no 632180) were transfected with pLVX‐IRES‐Hygro‐CD81‐Snorkel‐tag plasmid using Lenti‐X packaging single shots according to manufacturer instructions. In brief, 24 h before transfection, 4 × 10^6^ Lenti‐X 293 cells were seeded in a T75 flask in 8 mL growth media and incubated overnight at 37°C, 5% CO_2_. DNA was prepared for transfection by diluting 7 µg of pLVX‐IRES‐Hygro‐CD81‐Snorkel‐tag vector plasmid was diluted to 600 µL with sterile nuclease‐free water. Diluted plasmid vector DNA was added to Lenti‐X packaging single shots (TaKaRa; Cat.no: 631276) and vortexed for 20 s at high speed. Transfection mixture was incubated at room temperature for 10 min to allow nanoparticle complexes to form. After 10 min incubation, 600 µL of nanoparticle complex was added dropwise to 8 mL of cell culture prepared. Cells were incubated at 37°C, 5% CO_2_ overnight, 6 mL of fresh growth medium were added and incubate at 37°C, 5% CO_2_ for additional 48 h. After 48 h of incubation, lentiviral supernatants were harvested and filtered through 0.45 µm filter (VWR). The clarified supernatant was transferred to a fresh contained at a ratio of 3:1 with Lenti‐X concentrator (TaKaRa; Cat.no: 631232), mixed and incubated overnight at 4°C. Post‐incubation, sample were centrifuged at 1500 × *g* for 45 min at 4°C and the pellet was suspended gently in a tenth of the original volume of growth media. About 100 µL aliquots of virus stocks were prepared and stored in −80°C for future use.

For generating stable cells, target cells (HeLa and/or WJ‐MSC‐TERT 293 cells) were seeded in T75 flasks 18 h before transduction. Virus stock was thawed and mixed with growth medium containing polybrene (4 µg/mL) and added to the cells. After transduction, virus‐containing medium was replaced with normal growth media and cultured for 24 h. Then, medium was replaced with 20 µg/mL hygromycin containing fresh growth media and positive transformants were selected for 4–7 days. Once stable cells were generated, they were characterized and a master cell bank for future use was prepared.

### 2.3 Human platelet concentrates & plasma

Single donor platelet concentrates were provided by the Red Cross Blood Transfusion Service (Linz, Upper Austria). All samples were collected during routine thrombocyte apheresis in accordance with the policies of the Red Cross Transfusion Service, Linz. All blood donors signed their informed consent that residual blood material can be used for research and development purposes. All experimental protocols were approved by and carried out in collaboration with the Red Cross Blood Transfusion Service, Linz.

Platelet concentrates were generated by single platelet apheresis using an automated cell separator (Trima Accel Automated Blood Collection System, TerumoBCT) at the Red Cross Blood Transfusion Service (Linz, Upper Austria). All blood donors signed an informed consent that blood material can be used for research and the study was conducted in accordance with the policies of the Red Cross Transfusion Service. Platelets were finally stored in SSP+ (Macopharma) and ACD‐A (acid citrate dextrose + adenosine, Haemonetics^®^ anticoagulant citrate dextrose solution, Haemonetics^®^, Braintree) was used as an anticoagulant. About 2 mL of the platelet concentrate (containing ∼1 × 10^6^ platelets/µL) were aseptically transferred into a separate storage bag and experiments were carried out within 24 h after donation.

Plasma was provided by the Red Cross Blood Transfusion Service (Linz, Upper Austria). All samples were collected in accordance with the policies of the Red Cross Transfusion Service, Linz. All blood donors signed their informed consent that residual blood material can be used for research and development purposes. All experimental protocols were approved by and carried out in collaboration with the Red Cross Blood Transfusion Service, Linz.

### 2.4 EV isolation procedures

#### 2.4.1 Tangential flow filtration (TFF)

Conditioned media from HeLa‐WT and HeLa‐CD81‐Snorkel‐tag overexpressing cells were collected and subjected to a low‐speed spin at 700 × *g* for 5 min at 4°C to remove cellular debris, followed by 2000 × *g* spin for 10 min at 4°C to remove larger particles and cell debris. The supernatant was then sterile filtered with a 0.22 µm filters (VWR). Conditioned media was diafiltrated using two volumes of initial volume and concentrated to final volume of ∼35 mL using the KR2i TFF system (SpectrumLabs) using 300 kDa cut‐off hollow fibre filters (MidiKros, 370 cm^2^ surface area, SpectrumLabs) at a flow rate of 100 mL/min, a transmembrane pressure of 3.0 psi and a shear rate at 3700 s^−1^. Diafiltrate was further concentrated to ∼1 mL using Amicon ultra‐15 centrifugal filter unit (Catalogue # UFC910024) at 4°C with 3500 × *g*. Concentrated EVs were analysed for size and concentration by nanoparticle tracking analysis (NTA) and 100 µL aliquots were stored in PBS‐HAT buffer (Görgens et al., 2022) at −80°C for subsequent characterization studies.

#### 2.4.2 Ultrafiltration (UC)

Pre‐cleaned conditioned media (700 × *g* for 5 min and 2000 × *g* for 10 min) from HeLa‐WT and HeLa‐CD81‐Snorkel‐tag overexpressing cells was sterile filtered using syringe (VWR) with cellulose acetate membrane filters (0.22 µm pore size) to remove any larger particles. Then it was ultrafiltrated using 100 kDa MWCO Amicon ultra‐15 centrifugal filter unit (Catalogue # UFC910024) at 4°C with 3500 × *g*. Finally, the concentrates were diafiltrated with two volumes of 1X PBS and concentrated to final volume of ∼1 mL. Again, particles were quantified by NTA for size and concentration of EVs. After quantification EV sample were freshly used for further purification by StEVAC method.

#### 2.4.3 Ultracentrifugation (UF)

Human plasma EVs were isolated by ultracentrifugation as follows: 250 mL of fresh citrate plasma frozen at −25°C was provided by the Red Cross Blood Transfusion Service (Linz, Upper Austria, Austria). Plasma was thawed on ice, centrifuged twice at 2500 × *g* for 10 min and 5 mL of plasma were diluted 1:5 with PBS while the rest of the plasma was aliquoted into 10 mL volumes and stored at −80°C for future experiments. 1:5 diluted plasma was centrifuged at 14,000 × *g* for 30 min at 4°C. After centrifugation, supernatants were centrifuged at 100,000 × *g* at 4°C for 90 min using a P32ST swinging bucket rotor by ultracentrifuge CP100NX (Eppendorf, Germany). Supernatants were separated and pellets were resuspended in 1 mL of centrifuged supernatants to enrich for plasma EVs in plasma matrices.

For human platelet EV enrichment, fresh platelets from all the donors were incubated for 2 h on a shaker at room temperature (Burbidge et al., 2020). After incubation, samples were diluted 1:1. Platelets and cell debris were removed with a centrifugation for 5 min at 5000 × *g*, then at 14,000 × *g* for 30 min followed by 0.2 µm filtration. Filtered samples were immediately used for NTA, StEVAC and multiplex bead‐based flow cytometry (MBFCM) (Miltenyi Biotec, Germany).

### 2.5 Nanoparticle tracking analysis (NTA)

NTA was applied to determine particle size and concentration of all samples. All samples concentrated by TFF and UF were characterized by NTA with a NanoSight NS500 instrument equipped with NTA 2.3 analytical software and an additional 488 nm laser. Samples were diluted to 1:1000 in sterile filtered PBS (0.22 µm filter). Diluted samples were loaded in the sample chamber with camera level 13. Four to five 30 s videos were recorded per sample in light scatter mode with 5 s delays between each recording. Screen gain 10, detection threshold 7 were kept constant for all the recordings. Using the batch process option, all the measurements were analysed automatically.

### 2.6 Snorkel‐tag based extracellular vesicle affinity chromatography (StEVAC)

Conditioned media from HeLa‐WT and HeLa‐CD81‐Snorkel‐tag overexpressing cells were processed using ultrafiltration‐based EV isolation. Isolated EVs were quantified using NTA and ∼2.5 × 10^10^/mL were incubated with 250 µL of anti‐HA magnetic beads (Catalogue # 88836, ThermoFisher Scientific; bead concentration 10 mg/mL) overnight at 4°C on a rotospin test tube rotator. Post incubation, beads were separated on a magnetic rack, unbound EV solution was collected, and beads were washed with 0.22 µm filtered PBS. After washing, beads were resuspended in 0.22 mm filtered PBS with 1 mL (10 units) of PreScission protease (Catalogue # 27084301; GE healthcare Life Sciences) and incubated overnight at 4°C on a rotospin test tube rotator for on‐column PreScission protease cleavage. After overnight incubation, tubes were placed on a magnetic rack for separating beads and elutes were collected. Collected elutes along with flow through and washes were analysed for size and concentration using NTA and immediately used for characterization studies. For isolating EVs from pre‐cleaned supernatants, 45 mL of conditioned supernatants were incubated with 250 µL of anti‐HA magnetic beads overnight at 4°C on a rotospin test tube rotator. Post incubation, beads were spun down at 3000 × *g* for 10 min and flow through was collected. Beads were washed and incubated with PreScission protease (10 units) as described above. For pre‐mixed samples with HDF derived EVs, ∼2.5 × 10^10^/mL particles were mixed with equal number of HeLa‐CD81‐Snorkel‐tag enriched particles as inputs. For human platelet and plasma ∼5 × 10^10^/mL particles from human‐platelet or from plasma were mixed with equal number of HeLa‐CD81‐Snorkel‐tag EVs as inputs.

### 2.7 Immunoblotting

HeLa‐WT cells and HeLa‐CD81‐Snorkel‐tag expressing cells were collected and the cell pellet were lysed with 100 µL of RIPA buffer, kept on ice, and vortexed five times every 5 min. The cell lysate was then spun at 12,000 × *g* for 10 min at 4°C and the supernatant was transferred to a new tube and kept on ice. Protein concentrations for the supernatants were quantified by BCA assay (ThermoFisher Scientific) according to manufacturer's instructions. In brief, 50 µg of cell lysates and 1 × 10^9^ to 5 × 10^9^ particles were mixed with buffer containing 0.5 M dithiothreitol, 0.4 M sodium carbonate (Na2CO3), 8% SDS, and 10% glycerol, and heated at 95°C for 10 min. The samples were loaded onto a NuPAGE Novex 4%–12% Bis‐Tris Protein Gel (Invitrogen, Thermo Fisher Scientific) and run at 120 V in NuPAGE MES SDS running buffer (Invitrogen, Thermo Fisher Scientific) for 2 h. The proteins on the gel were transferred to an iBlot nitrocellulose membrane (Invitrogen, Thermo Fisher Scientific) for 7 min using the iBlot system. The membrane was blocked with Odyssey blocking buffer (LI‐COR) for 1 h at room temperature with gentle shaking. After blocking, the membrane was incubated overnight at 4°C or 1 h at room temperature with primary antibody solution (CD81, Santa Cruz Biotechnology, sc‐166029, 1:200; TSG101, abcam, ab125011, 1:1000; Alix, abcam, ab117600, 1:2000; Syntenin‐1, Origene, TA504796, 1:1000; Calnexin, abcam, ab22595; HA‐tag, Cell Signalling, 3724, 1:1000; SNAP/CLIP‐tag, NEB, P9310S, 1:1000; FLAG‐tag, Sigma, F3165, 1:5000). The membrane was washed with PBS supplemented with 0.1% Tween‐20 (PBS‐T, Sigma) three times for 5 min and incubated with the corresponding secondary antibody (LI‐COR; anti‐mouse IgG, 926‐68072, 1:10,000; anti‐rabbit IgG, 925–32213, 1:10,000) for 1 h at room temperature. Finally, the membrane was washed with PBS‐T for three times for 5 min, twice with PBS and visualized on the Odyssey infrared imaging system (LI‐COR) at 700 and 800 nm.

### 2.8 CLIP‐tag labelling quantification by flow cytometry

HeLa‐WT cells and HeLa‐CD81‐Snorkel‐tag overexpressing cells were collected and suspended in 1 mL of growth media (RPMI 1640 + 10% FCS + 1 X GlutaMAX). To this, non‐cell‐permeable CLIP‐substrate (CLIP‐SurfaceTM 647; Catalogue #S9234, NEB) was added with a final dilution of 1:100,000 and incubated for 1 h at 95% humidity, 5% CO_2_ and 37°C. Cells were spun at 300 × *g* for 5 min to remove the unlabelled dye, washed twice with PBS, pelleted at 300 × *g* for 5 min and resuspended in 100 µL of PBS. Dead cells were excluded by 4′,6‐diamidino‐2‐phenylindole (DAPI) staining and doublets were excluded by forward/side scatter area versus height gating. Samples were kept on ice and measured with MACSQuant Analyzer 10 flow cytometer (Miltenyi Biotec). GraphPadPrism 8.2.1 (GraphPadPrism Software, La Jolla, CA, USA) was used to analyse data and assemble figures.

### 2.9 Multiplex bead‐based flow cytometry assay for EV surface protein profiling

Different sample types were subjected to bead‐based multiplex EV analysis by flow cytometry (MACSPlex EV IO Kit, human, Miltenyi Biotec). Unless indicated otherwise, EV‐containing samples were processed as follows: samples were diluted with MACSPlex buffer (MPB) to, or used undiluted at, a final volume of 60 µL and loaded onto wells of a pre‐wet and drained MACSPlex 96‐well 0.22 µm filter plate before 3 µL of MACSPlex Exosome Capture Beads (containing 39 different antibody‐coated bead subsets) were added to each well. Generally, particle counts as quantified by NTA were used to estimate input EVs. We used 1 × 10^9^ particles as input EVs. Filter plates were then incubated on an orbital shaker overnight (14–16 h) at 450 rpm at room temperature protected from light. To wash the beads, 200 µL of MPB were added to each well and the filter plate was put on a vacuum manifold with vacuum applied (Sigma‐Aldrich, Supelco PlatePrep; −100 mBar) until all wells were drained. For counterstaining of EVs bound by capture beads with detection antibodies, 135 µL of MPB and 5 µL of each APC‐conjugated detection antibody cocktail (anti‐CD9, anti‐CD63, and anti‐CD81) were added to each well and plates were incubated on an orbital shaker at 450 rpm protected from light for 1 h at room temperature. Next, plates were washed by adding 200 µL MPB to each well followed by draining on a vacuum manifold. This was followed by another washing step with 200 µL of MPB, incubation on an orbital shaker at 450 rpm protected from light for 15 min at room temperature and draining all wells again on a vacuum manifold. Subsequently, 150 µL of MPB were added to each well, beads were resuspended by pipetting and transferred to V‐bottom 96‐well microtiter plate (Thermo Scientific). Flow cytometric analysis was performed using MACSQuant Analyzer 10 flow cytometer (Miltenyi Biotec). All samples were automatically mixed immediately before 70–100 µL were loaded to and acquired by the instrument, resulting in approximately 3000–5000 single bead events being recorded per well. FlowJo software (v10, FlowJo LLC) was used to analyse flow cytometric data. Median fluorescence intensity (MFI) for all 39 capture bead subsets were background corrected by subtracting the respective MFI values from matched non‐EV buffer (PBS) that was treated exactly like EV‐containing samples (buffer/medium + capture beads + antibodies) (Wiklander et al., 2018). GraphPadPrism 8.2.1 (GraphPadPrism Software, La Jolla, CA, USA) was used to analyse data and assemble figures.

For indirect labelling of Snorkel‐tag, samples were incubated with capture beads overnight (14–16 h) at 450 rpm at room temperature protected from light. The beads were washed with 200 µL MPB and 135 µL MPB added to each well and 15 µL of anti‐HA‐tag antibody (1:1000 final dilution) was added and incubated for 1 h at room temperature on an orbital shaker at 450 rpm protected from light. After incubation, the MPB in the wells was drained and wash steps were repeated. For counterstaining 15 µL of Dylight‐649 conjugated to anti‐rabbit detection antibody (1:1000 final dilution) was added and plates were incubated on an orbital shaker at 450 rpm protected from light for 1 h at room temperature. After incubation, MPB was drained, washed and flow cytometric analysis was performed using MACSQuant Analyzer 10 flow cytometer (Miltenyi Biotec) as mentioned above. MFI for all 39 capture bead subsets were background corrected by subtracting respective MFI values from matched non‐EV buffer (PBS) that were treated exactly like EV‐containing samples (buffer + capture beads + Dylight 649 detection antibody).

MBFCM EV quantification for flow throughs and elutes in the mixed human platelet EV and plasma EVs was performed according to manufacturer instructions. In brief, 5 × 10^9^ EVs/mL for inputs, 2.5 × 10^9^ EVs/mL for flow throughs and 2.5 × 10^8^ EVs/mL for elutes were subjected to bead‐based multiplex EV analysis by flow cytometry. For pre‐cleaned cell culture supernatant inputs and flow throughs, 120 µL of supernatants were used for capture. Flow‐cytometric analysis was performed using CytoFLEX S (Beckman Coulter). Data was analysed using CytExpert software (Beckman Coulter).

### 2.10 Flow cytometry of bead‐bound extracellular vesicles

For staining of EV tetraspanins and Snorkel‐tag on the EV surface, flow throughs and washes from StEVAC method were suspended in 20 µL of Exosome‐human CD81 flow detection beads (Thermo Scientific, 10622D) and incubated at 4°C overnight while rotating. The next day, bead‐bound EVs were separated using a magnetic rack. Bead‐bound EVs were suspended in 100 µL of PBS with antibody cocktail (CD63 Antibody, anti‐human, FITC, REAfinity™, 130‐118‐076; CD9 Antibody, anti‐human, APC‐vio770, REAfinity™, 130‐118‐813; anti‐FLAG, APC, REAfinity™, 130‐119‐584) (Miltenyi Biotec) and incubated at 4°C for 1 h while rotating. After staining, bead‐bound EVs were washed twice with PBS and suspended in 300 µL of PBS. Samples were kept on ice and data was acquired at a CytoFLEX S (Beckman Coulter). Data was analysed using CytExpert software. Median fluorescence intensity was normalized to the isotype controls (ΔMFI).

### 2.11 Fluorescence microscopy for cells

HeLa‐WT and HeLa‐CD81‐Snorkel‐tag overexpressing cells were seeded onto µ‐slides (ibidi GmbH, Martinsried, Germany) and incubated over night at 37°C. Then, cells were fixed with 4% paraformaldehyde for 15 min, washed two times with PBS, and permeabilized for 10 min in 0.3% Triton X‐100 followed by two PBS washes. Cells were blocked with 2% BSA prepared in PBS for 30–60 min. After blocking, slides were incubated in primary (HA‐tag, Cell Signalling, 3724, 1:800; CD81, Thermo Scientific, 11525542, 1:500) and secondary antibody (AF488, AF647, Jackson immunoResearch, 1:1000) solutions prepared in 2% BSA solution for 60 and 30 min, respectively in a humidified chamber at room temperature, each followed by three washes in PBS. Hoechst 33342 was included for counterstaining of DNA right before the last wash step.

For staining non‐permeabilized cells, cells were fixed and blocked in 2% BSA for 60 min and were directly incubated with primary and secondary antibodies as mentioned above.

### 2.12 Cellular uptake of StEVAC purified EVs

For quantification of cellular uptake of StEVAC purified EVs, equal number of eluted EVs from CD81‐Snorkel‐tag, WT and CD81‐Snorkel‐tag were labelled with CLIP‐substrate (CLIP‐Surface™ 647; Catalogue #S9234, NEB and/or CLIP‐Surface™ 488; Catalogue #S9232, NEB) with a final dilution of 1:100,000 was added and incubated for 1 h at 95% humidity, 5% CO_2_ and 37°C. Post‐incubation, unlabelled dye was removed by Zeba spin desalting columns (Thermo Fisher Scientific). Labelled EVs were added to human hepatocellular carcinoma cells (Huh‐7) seeded a day before at density of 3 × 10^4^ cells per well in a 96‐well plate. Cells were incubated for 2 h at 95% humidity, 5% CO_2_ and 37°C.

#### 2.12.1 By flow cytometry

After incubation, cells were washed twice with ice cold PBS, typsinized, spun down at 900 × *g* for 5 min and resuspended in 100 µL of PBS. Dead cells were excluded from analysis via 4′‐,6‐diamidino‐2‐phenylindole (DAPI) staining and doublets were excluded by forward/side scatter area versus height gating. Samples were kept on ice and data was acquired at a CytoFLEX S (Beckman Coulter). Data was analysed with CytExpert software. Mean fluorescence intensity was normalized over the control/untreated cell sample (ΔMFI)

#### 2.12.2 By fluorescence microscopy

After incubation, cells were washed twice with ice cold PBS. Hoechst33342 staining for nuclear counter staining. Next, cells were stained with lysotacker^®^ Green DND‐26 at 50 nM concentrations and analysed immediately with Leica TCS SP8.

### 2.13 Transmission electron microscopy (TEM)

For TEM analysis we used different protocols based on the sample and application.

All the solutions used for the staining procedure were pre‐filtered using 0.22 µm filters (VWR).

Freshly prepared EVs were adhered on Athene Old 300 mesh copper grids (Agar Scientific, Stansted, Essex, UK) and fixed with 1% glutaraldehyde. Grids were washed three times with nuclease free water (NFW) and stained for 5 min with 2% phosphotungstic acid hydrate (Carl Roth, Karlsruhe, Germany). The grids were left to dry and the specimens were visualized using TEM (FEI Tecnai T20, FEI Eindhoven, Netherlands) operated at 160 kV.

For all other TEM images, 5 µL of sample were added onto glow‐discharged formvar‐carbon type B coated electron microscopy grids for 3 min. Samples were removed by using wet Whatman filter paper. Grids were either prepared for immunogold labelling (see below) or carefully washed twice with filtered PBS. After washes, 5 µL of filtered 2% uranyl acetate were added for 10–30 s, uranyl acetate was removed using wet Whatman filter paper, grids were air dried and visualized using a transmission electron microscope (Tencai 10).

For immunogold labelling, grids were blocked after the initial binding step of the sample using filtered 2% BSA (in PBS) for 10 min. Primary and secondary antibodies were diluted in 0.2% BSA solution. After blocking, grids were placed on 15 µL primary antibody solution (anti‐CD81, 1:50; anti‐HA‐tag, 1:50) for 60 min. Post incubation, grids were washed with 0.2% BSA six times and incubated with secondary antibody (goat anti‐mouse secondary antibody conjugated with 10 nm gold particles and goat anti‐rabbit secondary antibody conjugated with 4 nm gold particle) in a dilution of 1:50 for 60 min. After incubation, grids were washed six times with PBS followed by six washing steps with ddH_2_O. Finally, grids were stained with 0.2% uranyl acetate for 10–30 s. Excess uranyl acetate was removed using wet Whatman filter paper, grids were air dried and visualized using a transmission electron microscope (Tencai 10).

### 2.14 EV RNA isolation

Freshly enriched EVs from WJ‐MSC WT and CD81‐Snorkel‐tag cell supernatants were immunoprecipitated either with hCD81 exosome isolation kit (Miltenyi biotec; 130‐110‐914) or µMACS™ HA Isolation Kit (Miltenyi biotec; 130‐091‐122). Trizol LS (Thermo Scientific) was added to EV precipitates. RNA was extracted by miRNeasy Mini Kit (Qiagen; 217004) and enriched for small RNAs by RNeasy MinElute Cleanup Kit (Qiagen; 74204) according to manufacturer instructions. The RNA integrity was tested on the Agilent 2100 Bioanalyzer using smallRNA kits (Agilent Technologies). RNA concentrations were calculated by using the Bioanalyzer software. For small RNAs this was calculated for lengths of 5–200 bp.

### 2.15 Library preparation and RNAseq

EV RNA samples were prepared for small RNA sequencing using QIAseq small RNA Library Prep kit (Qiagen), which includes the adapter ligation at the two ends of small RNAs, reverse transcription and amplification. During the reverse transcription, the unique molecular identifier (UMI) was added on each molecule. The quality of the libraries was checked on Agilent 2100 Bioanalyzer using High sensitivity DNA chips and the quantity of libraries was measured by qPCR using KAPA Library Quantification kit (Roche). Libraries were pooled using equal amount and sequenced on an Illumina Novaseq sequencer by single‐end 100 bp sequencing.

### 2.16 Bioinformatic analysis

The raw data was quality filtered and trimmed by fastx_toolkit, and adaptor sequences were removed using Cutadapt. The reads were collapsed to remove identical UMI‐containing reads. FastQC was used to ensure high‐quality sequencing data. The tRNA reads were filtered and the remaining reads were mapped to miRNAs from miRBase v22 using Bowtie allowing zero mismatches, but allowing for non‐templated 3′ A and T bases. Using the DESeq2 package in R, the small RNA expression profiles generated were used for differential expression analysis, and volcano plots were generated.

### 2.17 Statistical analysis

Statistics were either calculated with Excel or Graph Pad Prism, and respective tests are indicated below the figures in the result sections. Standard deviations were derived from at least three independent experiments. Two tailed tests were performed using an error probability of 0.05.

## 3 RESULTS

### 3.1 Design and validation of Snorkel‐tag

To design a versatile tag for a wide variety of applications for the EV field, several factors had to be carefully taken into consideration. We first selected an EV marker protein that should be ubiquitous to all EVs. For this reason, we chose the tetraspanin family members CD63 and CD81 as highly enriched in all classes of EVs^10^. Several caveats were carefully considered during the design. Importantly, the tag should be displayed on the EV surface and be versatile, in order to enable affinity purification and tracking of EVs. However, tetraspanins are multipass membrane proteins with N‐ and C‐termini located intravesicularly, and it was important to avoid inserting the tags into one of the extracellular loops of tetraspanins. This could produce unintended biochemical changes, leading to protein misfolding, instability, altered post‐translational modifications or aberrant enrichment within EV membranes, consequently leading to functional changes of the EVs (Maue, 2007; Park et al., 2009; Watschinger et al., 2008). Alternatively, omitting or adding transmembrane spanning regions is an option to have one of the termini displayed on the surface, however, again there is a risk of misfolding or wrong subcellular localization (Jürgen et al., 1997; Kohno & Igarashi, 2003). These considerations led us to generate a series of genetic constructs with CD63 and CD81 fusion proteins either lacking one transmembrane domain or by adding a fifth transmembrane domain. The additional domain was derived from mouse beta‐type platelet‐derived growth factor PDGFRB (Dowhan & Bogdanov, 2009) (Uniprot accession P05622) to display the respective N‐ or C‐terminus to the outside of the EVs (Figure S1), similar to a published snorkel construct (Brown et al., 2013).

**FIGURE 1:**
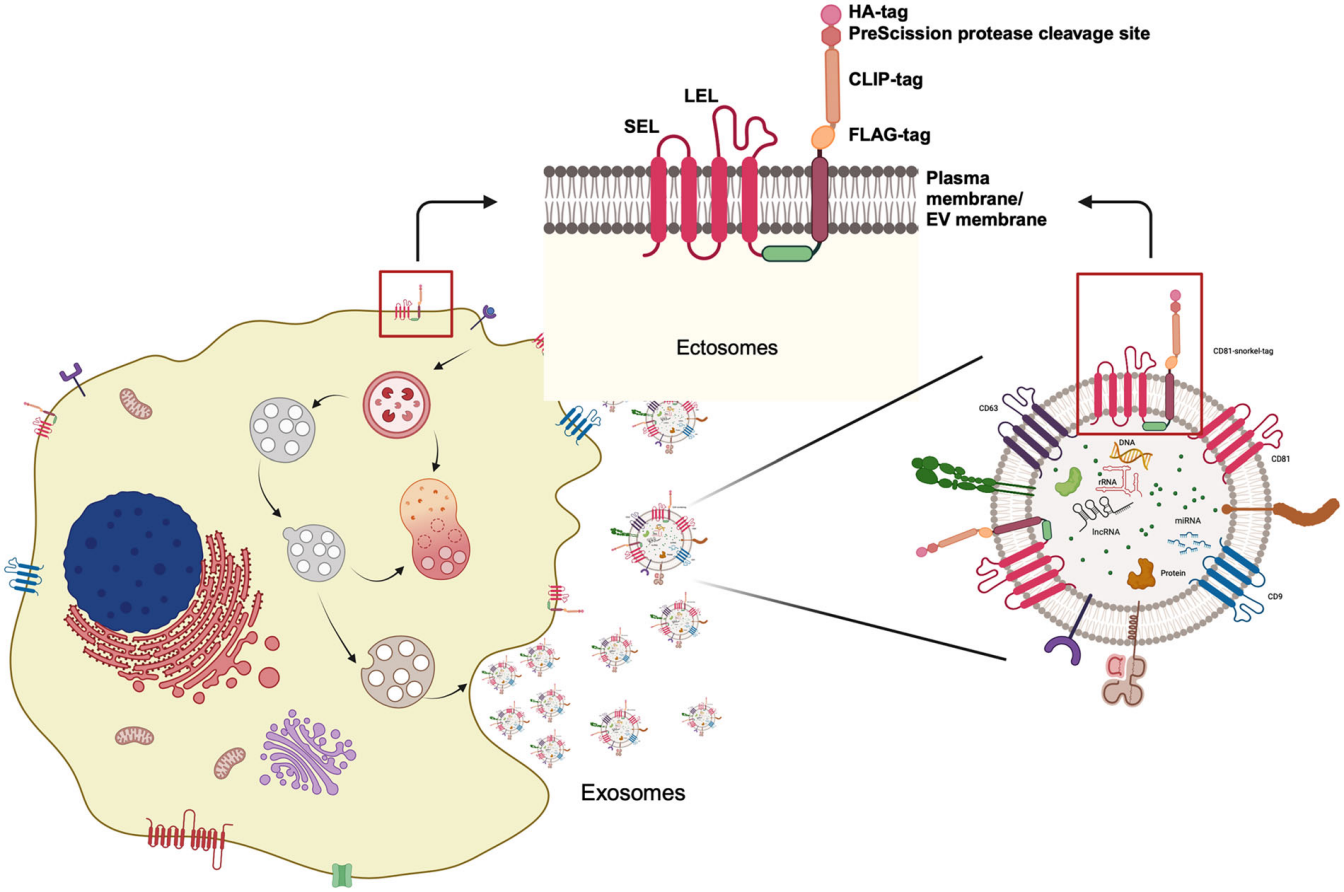
Schematic representation of the Snorkel‐tag genetically fused to the C‐terminus of CD81 on cell and EV surface membranes. Created with BioRender.com.

**FIGURE 2:**
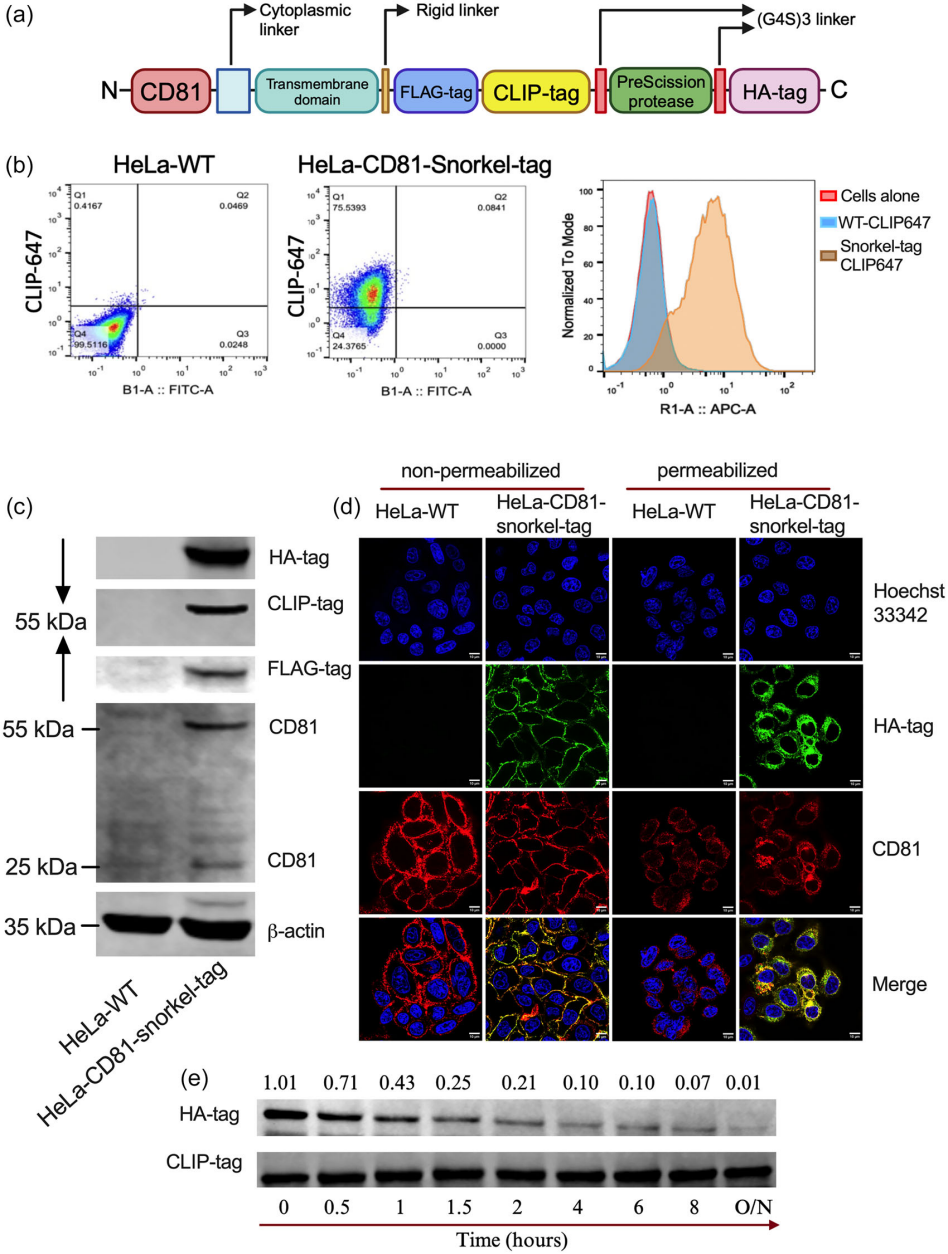
Expression of CD81‐Snorkel‐tag in HeLa cells results in accessibility of all parts of the Snorkel‐tag. (a) Schematic illustration of snorkel‐tag components fused to C‐termini of CD81. (b) Flow cytometric analysis of HeLa‐WT and HeLa‐CD81‐Snorkel‐tag stable cells stained with CLIP membrane impermeable substrate CLIP‐647. (c) Western blot of HA‐tag, FLAG‐tag, CLIP‐tag, CD81 and beta‐actin for WT and CD81‐Snorkel‐tag cell lines. Beta‐actin was used as loading control. (d) Fluorescent images of fixed WT and CD81‐Snorkel‐tag cells. Right two panels: Immunofluorescence (IF) for CD81 and HA‐tag in non‐permeabilized HeLa cells, Left two panels: IF for CD81 and HA‐tag in permeabilized HeLa cells. Counterstaining with Hoechst 33342 for DNA. (e) Western Blot of HeLa cells expressing CD81‐snorkel‐tag treated with PreScission protease for indicated time periods mentioned above. The blots were probed with antibodies against HA‐tag and CLIP‐tag. The mean intensity of the bands was quantified and the ratio of the HA‐tag versus CLIP‐tag is shown.

In this study, as a proof‐of principle, we selected the correctly membrane localizing fusion of CD81 (Figure S2) to a fifth transmembrane domain at its C‐terminus. In addition, the construct contains an additional extravesicular domain consisting of an HA‐tag and a PreScission protease cleavage site for non‐destructive elution from affinity columns. Further, a CLIP‐tag and a FLAG‐tag allow down‐stream staining with anti‐FLAG antibodies or covalent label with CLIP substrates. Finally, flexible linkers [(G4S)3] were introduced on either side of the PreScission protease to avoid steric hindrance of antibody binding and protease cleavage during affinity purification. This novel combined construct was termed Snorkel‐tag (Figures 1 and 2a). While we focused on CD81, we expect that any other tetraspanin molecule of EVs will be amenable to fusion with the Snorkel‐tag.

To produce recombinant Snorkel‐tag EVs, we stably expressed the CD81‐Snorkel‐tag construct in HeLa by the pLVX lentiviral transduction system. Stable cells were characterized for detectability of all Snorkel‐tag components, by detecting the CLIP‐tag in flow cytometry using a membrane impermeable fluorescent CLIP substrate (Figure 2b) and by immunoblotting for HA‐tag, FLAG‐tag and CLIP‐tag (Figure 2c). Confocal microscopy confirmed the localization of CD81‐Snorkel‐tag to the cell membrane on non‐permeabilized cells, and an intracellular localization in permeabilized cells (Figure 2d). Cleavability of the PreScission protease cleavage site was assessed and optimized by enzymatic digestion of whole cell lysates (Figure 2e). Taken together, these experiments confirm the expression of our Snorkel‐tag on the plasma membrane and its functionality and versatility for affinity purification and labelling.

### 3.2 Generation and characterization of EVs for Snorkel‐tag

**FIGURE 3:**
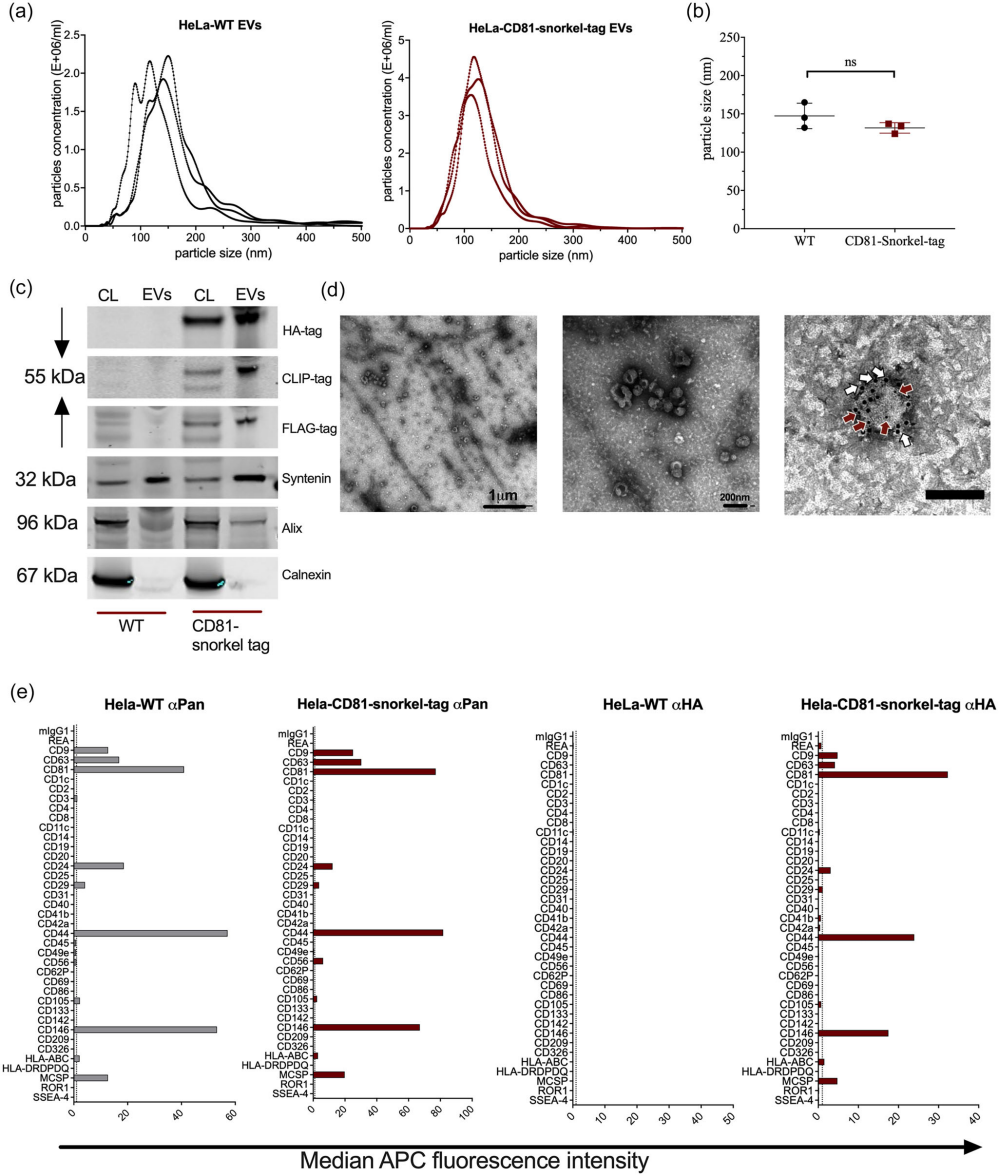
Snorkel‐tagged EVs isolated by tangential flow filtration (TFF) show similar characteristics as wild type EVs. (a) and (b) NTA analysis of TFF isolated EVs from HeLa‐WT and HeLa‐CD81‐snorkel‐tag for particle concentration and size. (c) Western blot of HeLa‐WT and HeLa‐CD81‐Snorkel‐tag cell lysates and EV lysates for Snorkel‐tag epitopes and EV specific markers. (d) Transmission electron microscopy (TEM) images for HeLa‐WT EVs and HeLa‐CD81‐Snorkel‐tag carrying EVs. Overview image (Left, scale bar 1 µm) and close‐up (right, scale bar 200 nm). White arrows label CD81 (anti‐mouse antibody tagged to10 nm gold particle) and red arrows label Snorkel‐tag (anti‐rabbit antibody tagged to 4 nm gold particle) (scale bar: 100 nm). (e) Multiplex bead‐based flow cytometry assay for detection of EV surface protein signature by pan tetraspanin detection antibodies and anti‐HA antibody.

Next, we aimed to validate the ultimate goal of non‐destructive affinity purification of EVs. For this, we used tangential flow filtration (TFF) to enrich EVs from conditioned media of HeLa‐CD81‐Snorkel‐tag and HeLa‐WT cells as controls. EV preparations were characterized for their concentrations and diameter by NTA (Figure 3a,b). TFF isolated EV preparations were enriched for Snorkel‐tag components (HA‐tag, CLIP‐tag and FLAG‐tag), classical EV‐specific markers (Alix, Syntenin‐1) and depleted from other intracellular proteins such as Calnexin, an endoplasmic reticulum specific protein (Figure 3c). Transmission electron microscopy (TEM) was used to validated the size and morphology of EVs in the expected range, as well as presence of CD81 and the HA‐tag by immunogold labelling (Figure 3d). We next evaluated if the EV protein surface signature would change due to CD81‐Snorkel‐tag overexpression using multiplex bead‐based flow cytometry (MBFCM) (Wiklander et al., 2018). EVs from both WT and CD81‐Snorkel‐tag overexpressing cells showed robust expression of abundant surface markers like tetraspanins, CD44, MCSP‐1 and CD146 with pan tetraspanin detection. As a negative control, we used an antibody targeting HA‐tag with Dylight‐649 secondary antibodies. As expected, we did not detect any background for WT EVs, while we did observe clear signals on CD81‐Snorkel‐tag enriched EVs for tetraspanins, CD44, MCSP‐1 and CD146. This indicates that the overexpression of CD81‐Snorkel‐tag did not alter the EV protein surface signature (Figure 3e). Together, these results provide evidence that the Snorkel‐tag fused to the C‐terminus of CD81 is well displayed on the surface of EV membranes.

### 3.3 Snorkel‐tag based extracellular vesicle affinity chromatography (STEVAC)

**FIGURE 4:**
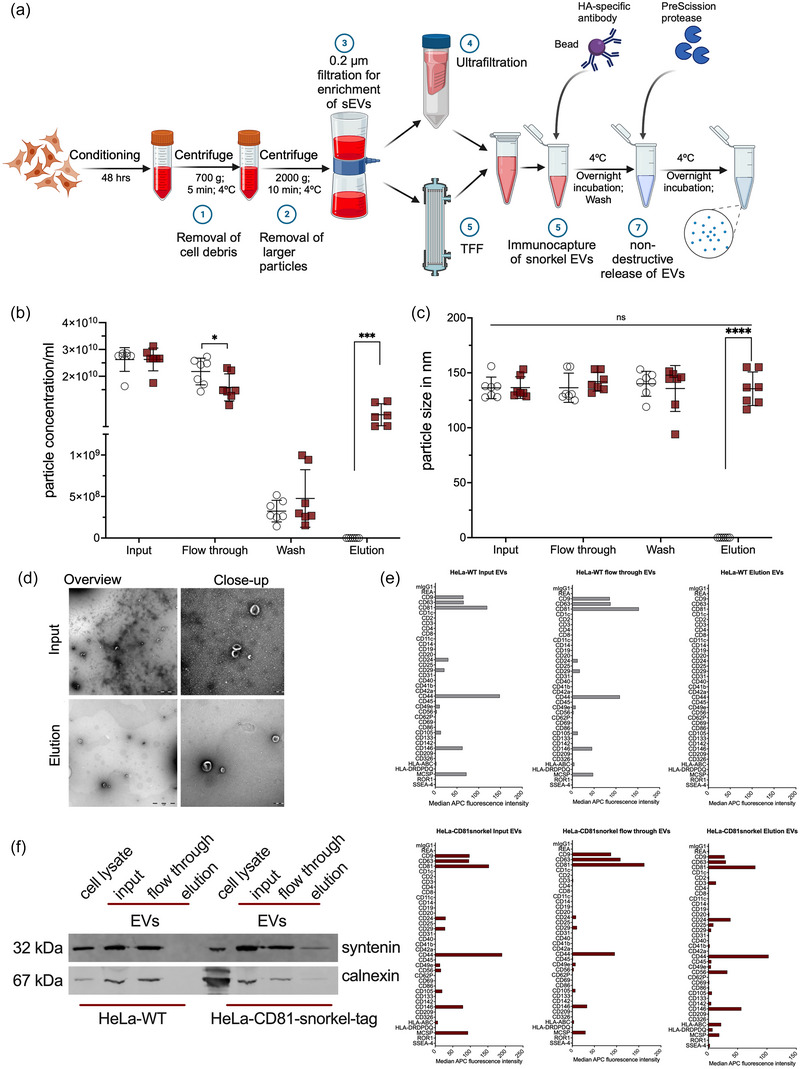
Snorkel‐tag based extracellular vesicle affinity purification. (a) Workflow for StEVAC method. Created with BioRender.com. (b) Nanoparticle tracking analysis (NTA) counts and (c) particle diameter of purified EVs eluted after incubation with PreScission protease overnight and a following wash step (*n* = 7). (d) Transmission electron microscopic examination for size and morphology of the input and eluted EVs (left panel with overview image, scale bar 1 µm; right panel with close‐up image, scale bar 200 nm). (e) Multiplex bead‐based flow cytometry assay to evaluate the StEVAC method. Assay results for HeLa‐WT (top) and HeLa‐CD81‐Snorkel‐tag (bottom); input, flow through (unbound EVs) and elution (*n* = 3). (f) Western blots for the EV‐associated protein syntenin and non‐EV marker calnexin (ER specific) in the eluates. One‐way ANOVA was applied on raw values; ns *p* > 0.05, **p < 0.01, ***p < 0.001.

To evaluate the purification of our recombinant EVs, we enriched them from conditioned media collected from HeLa‐CD81‐Snorkel‐tag cells and control HeLa‐WT cells. EVs were enriched by ultrafiltration using 100 MWCO Amicon filters, and the concentration and size were quantified by NTA (Figure S3). Next, we performed StEVAC on the enriched EVs. In brief, EVs were incubated with anti‐HA magnetic beads overnight at 4°C. Post incubation, beads were washed to remove uncaptured EVs and non‐specifically bound components. The captured EVs on the beads were then incubated with PreScission protease overnight at 4°C for on‐column cleavage (Figure 4a) and non‐destructive elution of EVs. This approach was chosen as an alternative to eluting with high salt or low pH buffers. While the latter is the common method used in affinity chromatography, the harsh conditions can destroy the EVs during the elution process.

StEVAC was performed in 3 to 7 independent replicates using ∼2.5 × 10^10^ particles/mL as input and wild type EVs as control. EVs in eluates were quantified by NTA showing a recovery of around 30% of input (Figure 4b), very similar to directly purifying the EVs from pre‐cleaned cell culture supernatants without prior enrichment (Figure S4). No differences in size profiles of unbound or eluted particles were observed (Figure 4c). Next, we performed a detailed characterization of StEVAC purified Snorkel‐tag EVs. EVs show the expected size in TEM, where we also observed a strong reduction of background signals (Figure 4d). MBFCM on flow throughs and eluates showed the same surface protein pattern as on input EVs, while WT eluates served as negative controls (Figure 4e), indicating the specificity of the method. Immunoblotting of the various fractions of StEVAC show de‐richment of calnexin as a marker for cytoplasmic contaminations (Figure 4f).

### 3.4 Confirming specificity of StEVAC

**FIGURE 5:**
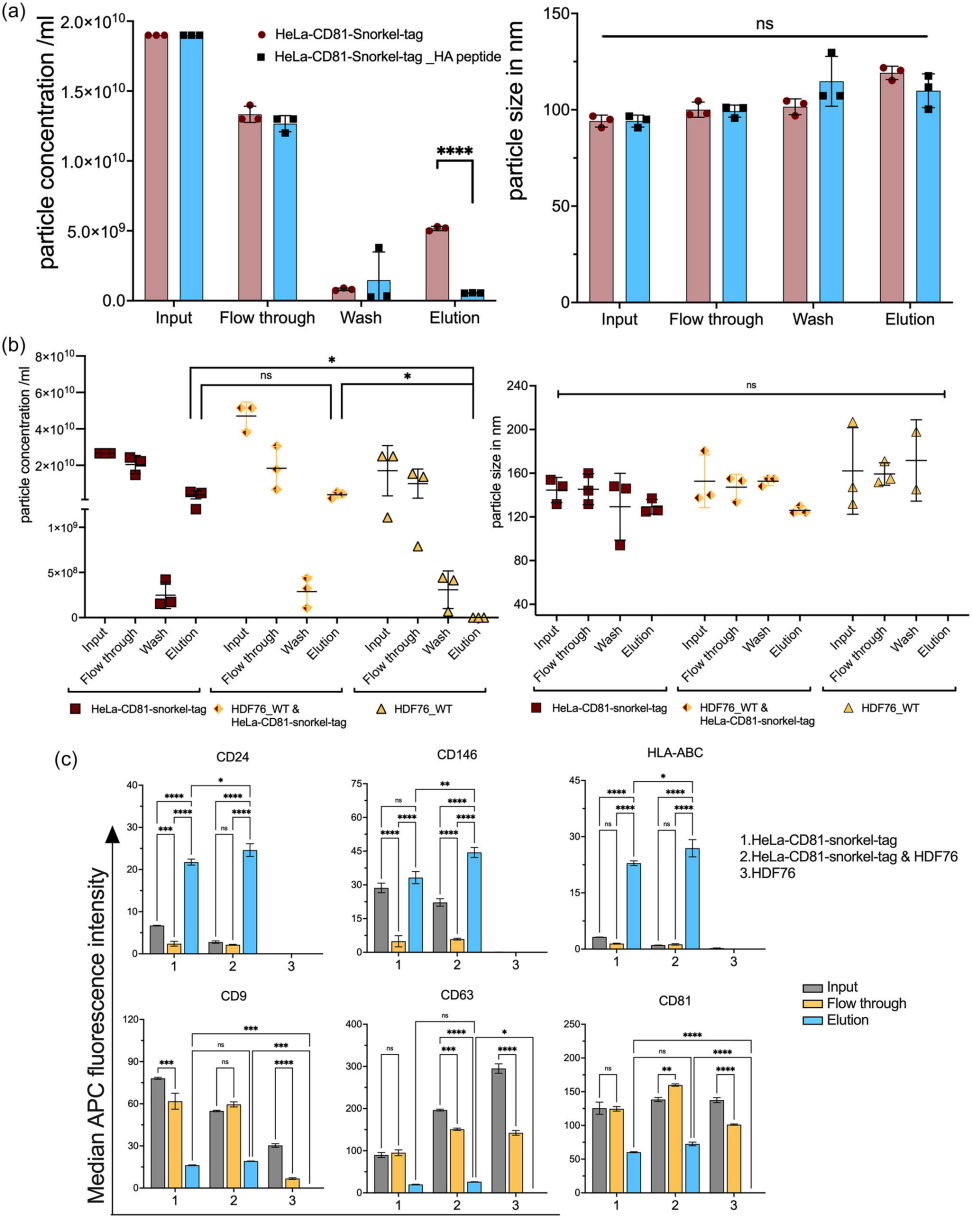
StEVAC specifically isolates snorkel‐tagged EVs out of complex mixtures. (a) Pre‐binding of anti‐HA matrix with HA peptide shows no binding of CD81‐Snorkel‐tag EVs henceforth no EVs in elution. Nanoparticle analysis (NTA) shows significant differences in elutions between anti‐HA matrix pre‐bound with HA peptide and free paratope (*n* = 3). (b) Nanoparticle tracking analysis (NTA) counts of purified EVs from HeLa‐CD81‐Snorkel‐tag, HDF76 and mix of both, eluted after incubation with PreScission protease treatment overnight and a following wash step (*n* = 3). No significant differences were observed in size distribution between flow through, washes and elution compared to input between CD81‐Snorkel‐tag EVs alone or mixed HDF76 EVs. (c) Multiplex bead‐based assay results for input, flow through and elution of EVs from HeLa‐CD81‐Snorkel‐tag, HDF76 and HeLa‐ CD81‐Snorkel‐tag mixed with HDF76. CD24, CD146 and HLA‐ABC are enriched only in the eluates of HeLa‐CD81‐Snorkel‐tag EVs (c; top row), additionally, tetraspanins CD9, CD63 & CD81 are enriched in all the elutes except in HDF76 (C; bottom row). Particle size quantification revealed no significant size difference during the isolation process (*n* = 3). One‐way ANOVA multiple comparison test was used for analysis of (a); 2‐way ANOVA multiple comparison test was used for analysis of (b) and (c). Data are shown as mean+_SD, *p < 0.05, **p < 0.01, ***p < 0.001.

We next confirmed the specificity of StEVAC by pre‐blocking anti‐HA magnetic beads with an excess of HA peptide before incubation with CD81‐Snorkel‐tag EVs. NTA results demonstrate that pre‐blocking the HA matrix with HA peptide indeed resulted in no recovery of EVs in the eluates (Figure 5a).

To test, if Snorkel‐tag EVs can be purified from EV mixtures, we combined HeLa‐CD81‐Snorkel‐tag EVs with primary human dermal fibroblast (HDF76) untagged EVs and performed StEVAC. We observed that the concentration of particles in the eluate from mixed EV inputs were similar to the CD81‐Snorkel‐tag EVs eluates (Figure 5b). Furthermore, we quantified diameter and EV protein signatures by multiplex bead‐based flow cytometry (MBFCM) before and after StEVAC. None of the 37 surface proteins included in the MBFCM panel were specific to fibroblast EVs, therefore, we had to rely on quantitating EV numbers and surface marker signal intensities of HeLa specific proteins CD24, CD146 and HLA‐ABC to estimate enrichment (Figures 5c and S5).

### 3.5 StEVAC method enables purification of Snorkel‐tag EVs from complex matrices

**FIGURE 6:**
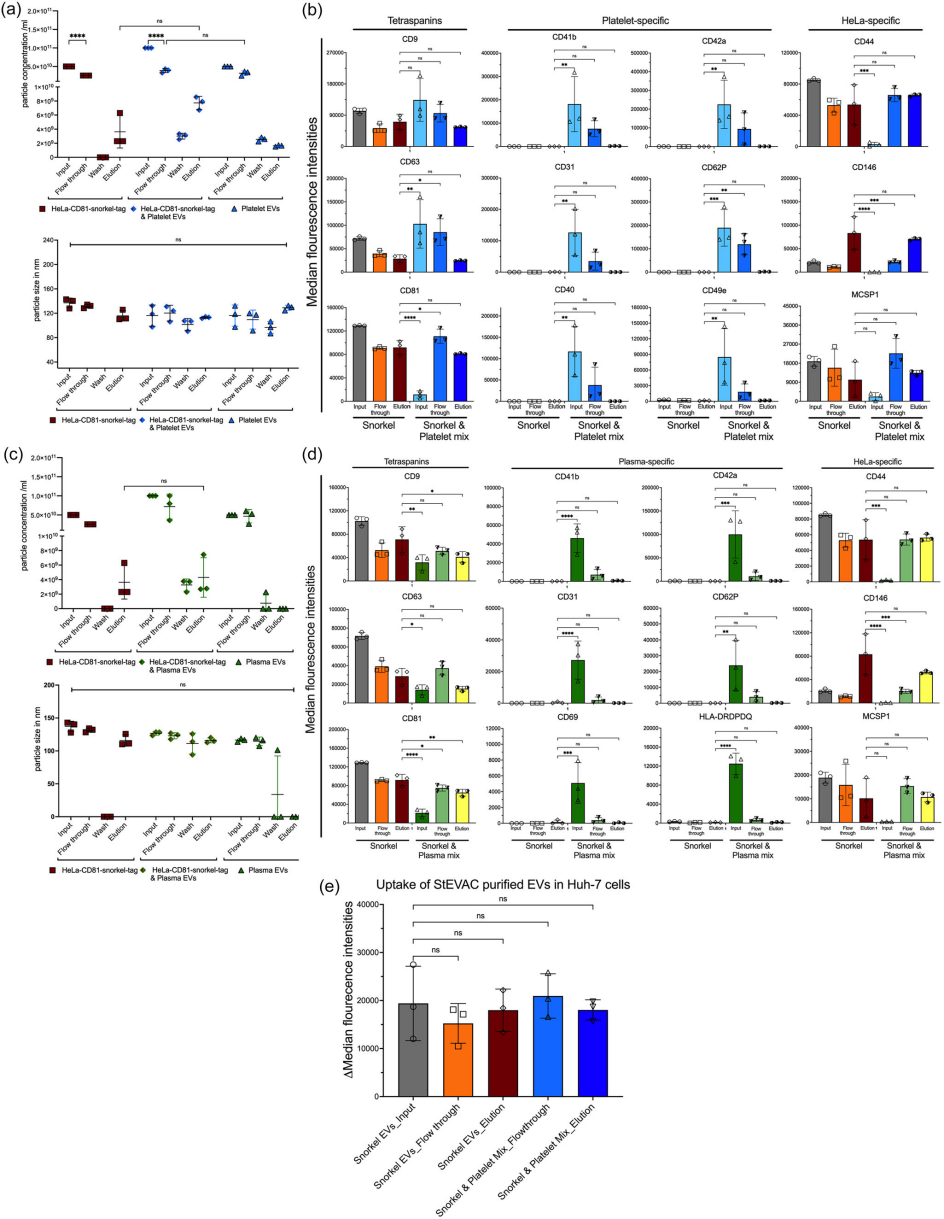
Confirming StEVAC for purification of EVs from complex matrices. StEVAC purification and characterization of HeLa‐CD81 Snorkel‐tag EVs from human platelet EV enriched mix. (a) Nanoparticle tracking analysis (NTA) counts of purified EVs from HeLa‐CD81‐Snorkel‐tag and mix of human platelet EVs (*n* = 3 individual donors), eluted after incubation with PreScission protease treatment overnight and a following wash step. (b) Multiplex bead‐based flow cytometry (MBFCM) assay results for input, flow through and elution of EVs from HeLa‐CD81‐Snorkel‐tag and HeLa‐ CD81‐Snorkel‐tag mixed with human‐platelet EVs. CD44, CD146 and MCSP1 are enriched only in the elutes for HeLa‐CD81‐Snorkel‐tag EVs (right column), platelet EV specific CD31, CD41b, CD42a, CD62P, CD40 and CD49e are depleted (centre 2 columns). StEVAC purification and characterization of HeLa‐CD81 Snorkel‐tag EVs from human plasma mix. (c) Nanoparticle tracking analysis (NTA) counts of purified EVs from HeLa‐CD81‐Snorkel‐tag and mix of human platelet EVs, eluted after incubation with PreScission protease treatment overnight and a following wash step (*n* = 3). (d) Multiplex bead‐based flow cytometry (MBFCM) assay results for input, flow through and elution of EVs from HeLa‐CD81‐Snorkel‐tag and HeLa‐ CD81‐Snorkel‐tag mixed with human‐plasma EVs. CD44, CD146 and MCSP1 are enriched only in the elutes for HeLa‐CD81‐Snorkel‐tag EVs (right column), plasma EV specific CD31, CD41b, CD42a, CD62P, CD69 and HLA‐DRDPDQ are de‐riched (centre 2 columns). Tetraspanins CD9, CD63 and CD81 are enriched equally in both the elutes. (e) Flowcytometric analysis of uptake StEVAC purified EVs from mixture of platelet concentrates labelled with CLIP‐505 substrate into Huh‐7 cells. Unpurified EVs (or) input EVs and flow through EVs were used as control (*n* = 3). Same number of particles were used for controls and elutes in uptake. One‐way ANOVA was applied on values; ns *p* > 0.05, *p < 0.05, **p < 0.01, ***p < 0.001. 2‐way ANOVA multiple comparison test was used for analysis of (a) and (c). One‐way ANOVA multiple comparison test was used for analysis of (b), (d) and (e). Data are shown as mean + SD, *p < 0.05, **p < 0.01, ***p< 0.001.

Next, we tested the potential of StEVAC to affinity purify Snorkel‐tag EVs from more complex matrices such as human platelet derived EVs and plasma as a surrogate for *ex vivo* purification from preclinical models. We mixed HeLa‐CD81‐Snorkel‐tag EVs enriched by ultrafiltration with human platelet EV concentrates from three different donors pre‐cleaned by differential centrifugation to remove platelets and large EVs. StEVAC purified EVs from mixed populations show similar concentrations compared to HeLa‐Snorkel‐tag EV eluates (Figure 6a) and the eluates showed HeLa EV specific surface proteins CD44, CD146 and MCSP1 and depletion of platelet EV markers CD31, CD41b, CD42a, CD62P, CD40 and CD49e (Figures 6b and S6A). Next, we mixed human plasma EVs isolated by differential ultracentrifugation with three individual HeLa‐Snorkel‐tag EV concentrates enriched by ultrafiltration. Indeed, NTA results showed no significant differences in particle concentration in eluates from control and plasma mixed EVs (Figure 6c). MBFCM showed enrichment for HeLa EV‐specific surface proteins and depletion of plasma EV markers CD31, CD41b, CD42a, CD62P, CD69 and HLA‐DRDPDQ (Giovanazzi et al., 2023) (Figures 6d and S6B, Karimi et al., 2022). In addition, StEVAC purified EVs are actively taken up into Huh‐7 cells at similar efficiency as freshly enriched EVs (Figures 6e and S7). This suggests that Snorkel‐tag EVs can be isolated from complex sample matrices, paving the ground for better understanding of EV cargo under various conditions.

### 3.6 Snorkel‐tag EV miRNA cargo is highly similar to wild type EVs

**FIGURE 7:**
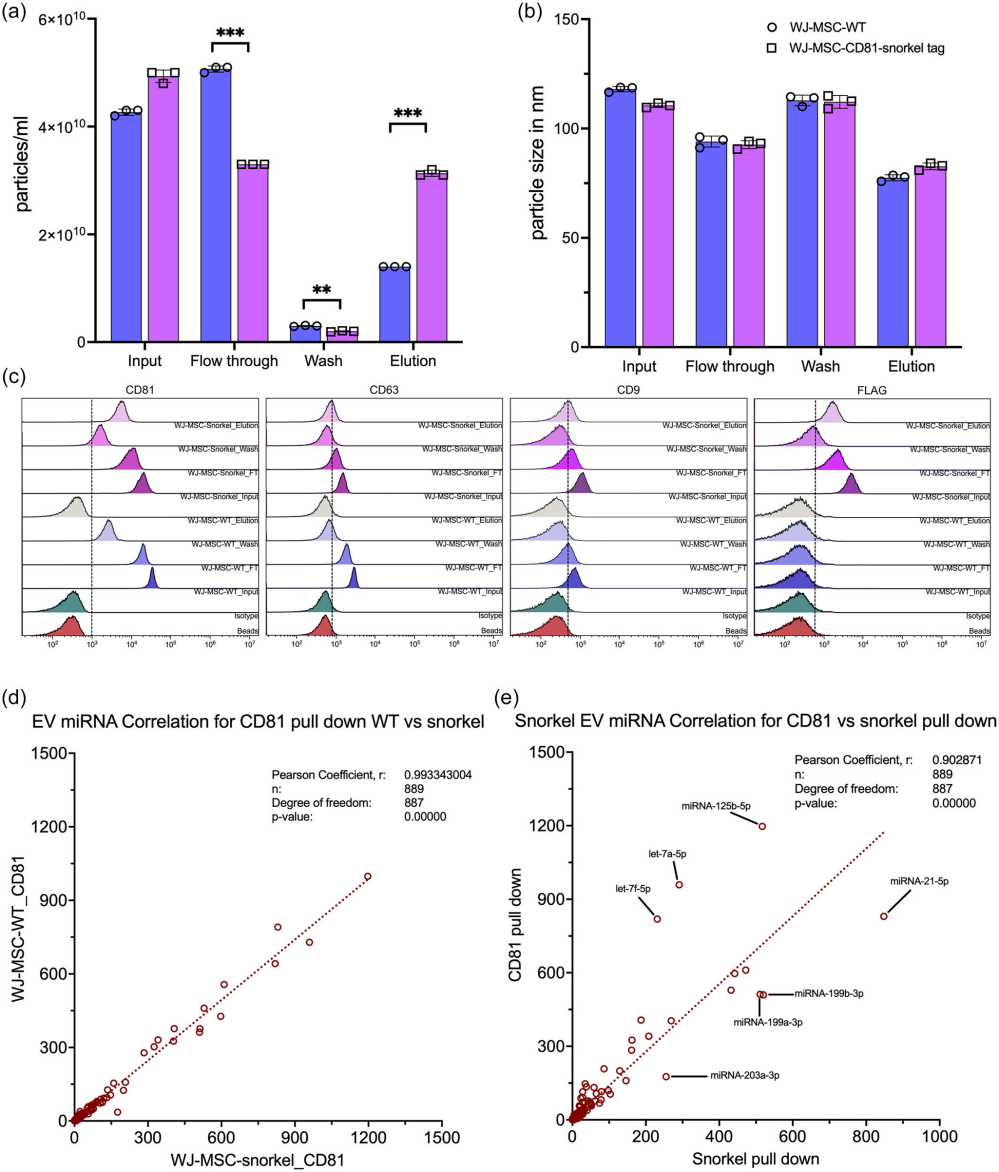
Influence of Snorkel‐tag on EV cargo loading. StEVAC purification of EVs from WT and CD81‐Snorkel‐tag WJ‐MSCs. (a) Nanoparticle analysis (NTA) shows significant differences in elutaes of WT versus CD81‐Snorkel‐tag EVs. (b) no significant differences were observed in size distribution between flow through, washes and elution compared to input. (c) Bead‐based flow cytometry evaluation of flow throughs, washes and eluates from WJ‐MSC‐WT and WJ‐MSC‐CD81‐Snorkel‐tag EVs for CD63, FLAG‐tag and CD9. (d) Pearson correlation scatter plots of miRNAs expression levels between WJ‐MSC derived EVs isolated by anti‐CD81 pull‐down from CD81‐Snorkel‐tag enriched EVs and WT EVs (*n* = 3). (e) Pearson correlation scatter plots of miRNAs expression levels between WJ‐MSC derived CD81‐Snorkel‐tag enriched EVs purified by either CD81 specific pull‐down or by Snorkel‐tag pull down shows only minor differences in cargo loading (*n* = 3).

In addition to HeLa cells, CD81 Snorkel‐tag construct was introduced into telomerised MSCs, WJ‐MSC/TERT273 cells, as an example of a therapeutically relevant EV origin, by lentiviral transduction and selected for CD81‐Snorkel‐tag expression. Stably expressing WJ‐MSC/TERT273 cells showed typical specific markers CD73, CD90 and CD105 (data not shown). Conditioned media from WJ‐MSC/TERT273 WT and CD81‐Snorkel‐tag cells were concentrated by 100 MWCO Amicon filters and StEVAC was performed. NTA results demonstrate ∼30% of Snorkel‐tag EVs in the eluate (Figure 7a). However, we observed a slight background signal from the WT eluates with no significant differences in diameter of the particles (Figure 7b). To evaluate the purity of eluates, we performed bead‐based flow cytometry by staining CD9, CD63, CD81 and FLAG on flow compatible CD81 magnetic beads. In contrast to NTA results, we observed strong signals for tetraspanins and FLAG in Snorkel‐tag eluates and no signals in the elutaes from WT EVs (Figure 7c). These results strongly suggest a high specificity of StEVAC also in the context of MSC‐EVs, with the additional tags allowing for tracking experiments.

To elucidate if the overexpression of the CD81‐Snorkel‐tag alters the composition of miRNAs which are associated with MSC‐EVs, EVs from WJ‐MSC‐CD81 Snorkel‐tag versus wild type cells were immunoprecipitated by anti‐CD81 antibodies was performed using 3 independent purifications per group. Small RNA sequencing was performed to analyse miRNAs, and all other, more abundant RNA species, including mRNA, rRNA, tRNA, snoRNA, and snRNA were filtered from the total raw reads. A total of 889 miRNAs were identified in all samples with an almost perfect correlation (Pearson *r* ^2 ^= 0.99) and no miRNA differences reaching statistical significance (Figure 7d and S8C). Similarly, we compared the miRNA cargo of Snorkel‐tagged EVs immunoprecipitated by anti‐HA versus anti‐CD81 antibodies resulting in a very high correlation (Pearson *r* ^2 ^= 0.90), with only 7 out of 889 (0.78%) miRNAs being significantly differentially present (Figures 7e and S8D). An overview of total reads from nine libraries is shown in Figure S8A. Out of approximately 20 million reads per sample, miRNAs showed less than 10% enrichment in EVs (Figure S8B). These results again suggest that stable Snorkel‐tag overexpression leads to only minimal differences in the composition of EVs, making it the ideal tool to analyse endogenous EV expression and behaviour.

## 4 DISCUSSION

Recent advances in nanoparticle isolation and analytics have led to the identification of ever‐increasing numbers of different EV subtypes and non‐vesicular extracellular nanoparticles (NVEPs) such as lipoproteins, ribonucleoproteins or recently discovered exomeres (Zhang et al., 2018, 2019) and super meres (Jeppesen et al., 2022; Winter et al., 2021; Zhang et al., 2021). This increasing complexity of EV  heterogeneity has been reflected by the updated MISEV 2023, consensus position paper, in which a pragmatic approach for EV nomenclature has been taken by classifying EVs < 200 nm as small EVs (sEVs) or >200 nm as large EVs (lEVs) even irrespective of their biogenesis (Welsh et al., 2024). With such complexity and heterogeneity, purification strategies capable of specifically isolating single EV species are of high need to define their functionality.

So far, various EV enrichment methods have been developed. Differential ultracentrifugation (dUC) remains as the most common, providing relatively high purity but bringing with it limitations in scalability and co‐precipitation of protein aggregates (Driedonks et al., 2019; Sódar et al., 2016). Ultrafiltration and TFF are alternatives offering less harsh conditions as compared to dUC and are more easily scalable (Corso et al., 2017). Size exclusion chromatography has emerged as a leading method to achieve EVs without co‐contaminants from biological fluids or when combined with dUC or TFF (Karimi et al., 2018), however it is unclear at this point, if this also removes bioactive EV molecules residing in the EV corona (Bellotti et al., 2021; Wei et al., 2020). Immunoprecipitation is another alternative method for EV isolation from biological fluids that provides high specificity towards discrete EV subpopulation. However, the choices of antibodies is critical (Fortunato et al., 2022) and elution after capture is difficult to achieve without destroying the integrity of EVs as usually high salt above 1 M leading to osmotic disruption or pH below three of the eluent is necessary to unbind the EVs from affinity matrix. Taken together, EV heterogeneity, co‐isolation of contaminants, EV integrity and cargo preservation, functional assay compatibility, standardization and reproducibility are bottlenecks in understanding the mode of action of EVs, hindering the development of therapeutics (Li et al., 2023). Similarly, reference materials and standards of specific EVs are necessary to define their intrinsic heterogeneity, source and recovery efficiency in EV separation methods (Geeurickx et al., 2021; Welsh et al., 2020). Recent reports on heparin‐, and aptamer‐, tim4‐based affinity purifications showed an increased purity of EVs (Balaj et al., 2015; Barnes et al., 2022; Nakai et al., 2016; Zhu et al., 2023), however, detailed investigations on EV functionality and co‐isolation of glycoproteins are needed to confirm this (Balaj et al., 2015; Barnes et al., 2022; Reiter et al., 2019).

To address these limitations in the field, we developed a Snorkel‐tag for the non‐destructive isolation of EVs using extracellular vesicle affinity chromatography (StEVAC). CD81 is the most highly enriched EV membrane protein in almost all subtypes of EVs (Lischnig et al., 2022). On cell surfaces, CD81 plays an important role in adaptive immunity by its interaction with CD19 and CD21 in B cell receptor signalling (van Zelm et al., 2010). It has been shown that mutations in CD81 resulted in disruption of CD19 complex formations on B cell leading to antibody deficiency syndrome in humans (Mittelbrunn et al., 2011). Additionally, the transmembrane domain (TM1) and large extracellular loop (LEL) of CD81 play an important role in trafficking, localization of co‐receptors and signalling (Levy, 2014; Matsumoto et al., 1993; Mattila et al., 2013; Susa et al., 2020; van Zelm et al., 2010; Witherden et al., 2000). Considering the widespread presence of CD81 on various EVs, we genetically fused it to the Snorkel‐tag, specially designed for transmembrane proteins such as GPCRs or ion‐channels where both N‐ and C‐ termini of proteins are inside the cell. This also warrants that the Snorkel‐tag is spatially separated from CD81 by linker sequences and does not interfere with CD81 functionality. However, we point out, that we did not investigate if signalling functions due to the protein engineering would have been disturbed, as we focused on using the genetically encoded CD81‐Snorkel‐tag fusion protein that displays the Snorkel‐tag components on the surface of EV membranes to allow for easy isolation and covalent labelling of EVs *in vitro*.

To expand the application of our novel tagging system, we then systematically evaluated StEVAC as a method for purifying EVs harbouring the Snorkel‐tag directly from cell culture supernatants, by concentrating the culture supernatants after ultrafiltration followed by rigorous characterizations, or from complex matrices when spiked into multiple biofluids. Indeed, we purified Snorkel‐tag harbouring EVs from plasma EVs and platelet concentrates without non‐Snorkel‐tagged EV contaminants. These examples illustrate the wide‐spread use cases *in vitro*, *ex vivo* or even *in vivo*.

To provide mechanistic insights into EV uptake, biodistribution, and pharmacokinetics, it is a pre‐requisite to reliably track EVs *in vitro* and *in vivo*. Several labelling approaches currently in use, rely on fluorescent or bioluminescent reporters, or on substances compatible for imaging. Caveats associated with genetic fusion may affect the subcellular localization and function of proteins. Additionally, due to high background and low tissue penetration of signals, these methods reveal little about EV trafficking. To overcome these limitations, we introduced the CLIP‐tag as a component of the Snorkel‐tag, which is a covalent self‐labelling protein‐tag that can be specifically and irreversibly labelled with O^2^‐benzyl‐cytosine (BC‐) derivatives. Using the CLIP‐tag has the advantage that the majority of model organisms does not react with benzyl‐cytosine derivatives, avoiding any endogenous activity and background (Gautier et al., 2008). Furthermore, a number of commercially available cell‐permeable and impermeable BC fluorophores can be used not only *in vitro* or after EV purification, but also on fixed tissue samples, avoiding the issue of disrupting EV biodistribution with the dyes. Despite the Snorkel‐tag EVs displaying retained uptake behaviour *in vitro*, future studies need to be performed to assess the impact of the tag on the *in vivo* distribution of EVs.

**FIGURE 8:**
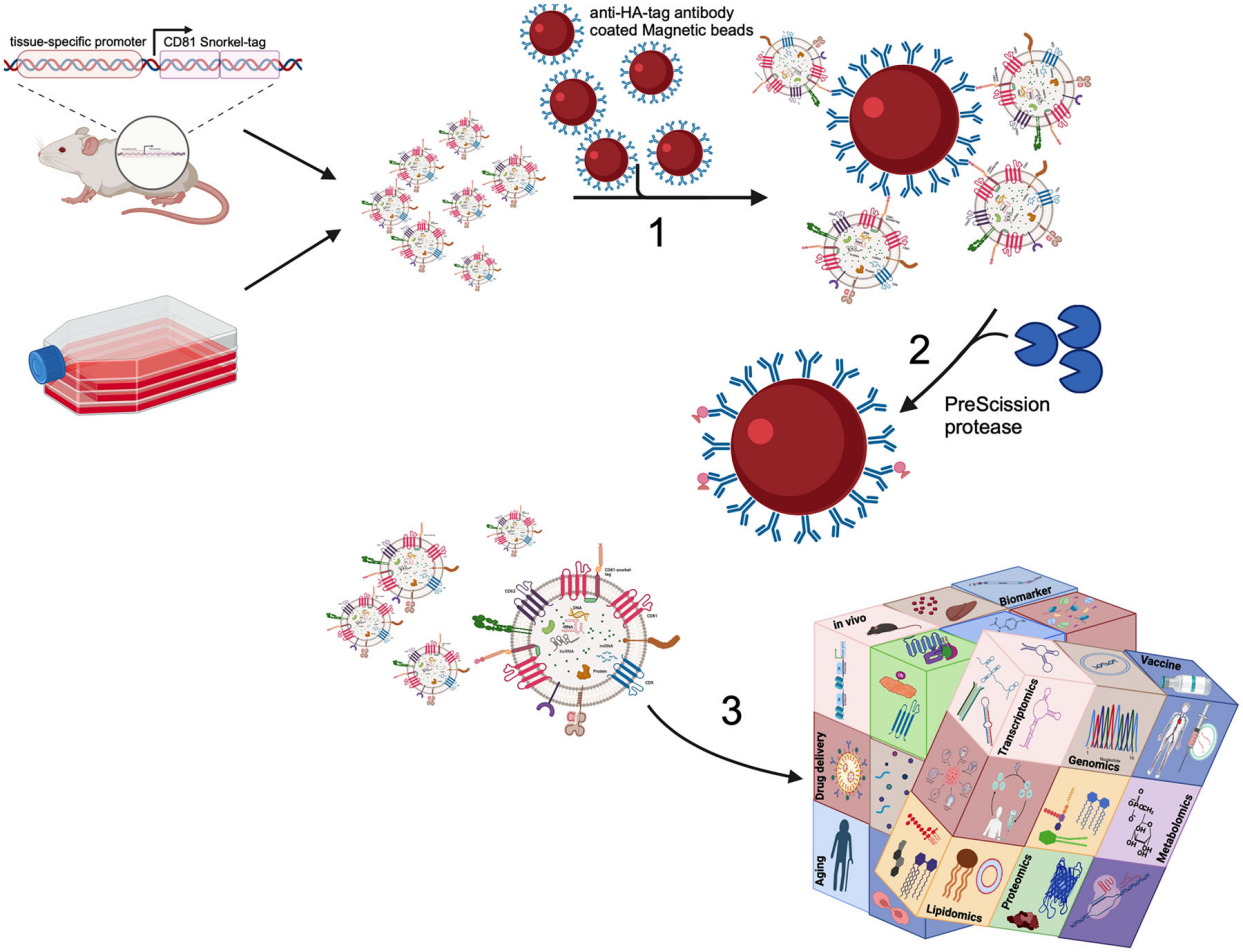
The StEVAC platform. Overview on Snorkel‐tag based EV affinity chromatography (StEVAC): (1) CD81‐Snorkel‐tag enriched EVs from *ex vivo* or *in vivo*, incubated overnight with anti‐HA matrix at 4°C to capture Snorkel‐tag enriched EVs. (2) Mild treatment of captured EVs with PreScission protease at 4°C releases EVs without changing their biophysical properties. (3) StEVAC method enables to understand the cargo of EVs under normal and disease physiological states, when expressed under tissue‐specific promoter in transgeneic mouse models. Created with BioRender.com.

In summary, our results in any case demonstrate that the Snorkel‐tag and subsequent StEVAC is a fast methodology, allowing for the tracking of EVs upon uptake into cells *in vitro*. These results indicate that this methodology is likely to have high success rates when it is expanded to *in vivo* studies (Monroe et al., 2024). As summarized in Figure 8, this technology can drive the understanding of EV biology, contribute to the simplified production of reference material for EV research, and thus advance the development of EV‐ based therapeutics and diagnostics.

### 4.1 Data resources

The accession number for the RNA‐seq reported in this paper is GEO: GSE251842. The CD81‐Snorkel‐tag fusion protein has been submitted to GenBank under the accession number PQ063272.

## AUTHOR CONTRIBUTIONS

Conceptualization, Madhusudhan Reddy Bobbili, André Görgens, Jorgen Kjems, Samir EL Andaloussi, Johannes Grillari; Methodology, Madhusudhan Reddy Bobbili, André Görgens, Yan Yan, Matthias Hackl, Jaroslaw Jacak, Markus Schosserer, Regina Grillari; Experiments performed by Madhusudhan Reddy Bobbili, André Görgens, Yan Yan, Stefan Vogt, Dhanu Gupta, Giulia Corso, Samir Barbaria, Carolina Patrioli, Sylvia Weilner, Marianne Pultar; Madhusudhan Reddy Bobbili wrote the manuscript; Jorgen Kjems, Samir EL. Andaloussi and Johannes Grillari supervised the project. All authors contributed to the article and approved the submitted version.

## CONFLICT OF INTEREST STATEMENT

Johannes Grillari and Regina Grillari are co‐founder and shareholder of Evercyte GmbH. Matthias Hackl serves as a CEO and co‐founder, Johannes Grillari and Regina Grillari are co‐founders and serves on the scientific advisory board of TAmiRNA GmbH. André Görgens, Dhanu Gupta and Samir EL Andaloussi are consultants for and have equity interests in Evox Therapeutics Ltd., Oxford, United Kingdom. All other authors declare no potential conflict of interests.

## Supporting information



Supplementary figure 1. (A) Schematic representation of full length CD81 genetically fused with Snorkel‐tag at C‐termini and CD81 truncated versions devoid of either transmembrane domains 1 or 4 with Snorkel‐tag fused to SEL (small extracellular loop) or LEL respectively. (B) Western blot of anti‐HA tag for all four fusion proteins transiently expressed in HeLa cells. Western blot results reveal N‐terminal truncated version of CD81 snorkel‐tag did not express full length protein. Created with BioRender.com.

Supplementary figure 2. Fluorescent images of fixed HeLa cells expressing CD81 with N‐terminal Snorkel‐tag and C‐terminal truncated Snorkel‐tag stained with anti‐HA tag antibody and Alexafluor‐488 anti‐rabbit secondary antibody.

Supplementary figure 3. (A) Representative particle size and concentration for EVs derived from HeLa‐WT and HeLa‐CD81‐Snorkel‐tag cell lines (*n* = 3). (B) Particle concentrations and size of ultrafiltrated particles from 75 mL conditioned media from 6 individual experiments. Unpaired t‐test was applied on raw values; nsP > 0.05.

Supplementary figure 4. Purification of Snorkel‐tag harbouring EVs from pre‐cleaned supernatants by StEVAC. NTA counts and particle diameter of purified EVs from supernatants (A) and (B). Multiplex bead‐based assay results for input, flow through, concentrated flow through and elution of EVs from HeLa‐WT (C) and Hela‐CD81‐Snorkel‐tag (D). 1‐way ANOVA was applied on raw values; nsP > 0.05, **p < 0.01, ***p < 0.001.

Supplementary figure 5. Confirming StEVAC to purify EVs carrying Snorkel‐tag from mixed population of EVs. Multiplex bead‐based assay results for input, flow through and elution of EVs from HeLa‐CD81‐Snorkel‐tag (A); HDF76 (B) and HeLa‐ CD81‐Snorkel‐tag mixed with HDF76 (C).

Supplementary figure 6. MACSPlex 37 EV surface protein panel for HeLa‐CD81‐Snorkel‐tag and in mixtures with human platelet (A) and with human plasma (B); inputs, flowthroughs and elutes probed by anti‐pan tetraspanin APC antibodies (*n* = 3).

Supplementary figure 7. Uptake of StEVAC purified EVs in Huh‐7 recipient cells. Representative Confocal images of StEVAC purified EVs labelled with CLIP‐647 uptake in Huh‐7 cells from HeLa‐WT, HeLa‐CD81‐Snorkel‐tag, as a positive control HeLa‐CD81‐Snorkel‐tag unpurified EVs in red. Counter staining with LysoTracker in green.

Supplementary figure 8. EV cargo characterization. (A) total reads of small RNA sequencing from WJ‐MSC WT and Snorkel‐tag EVs pull down from Snorkel‐tag and CD81. (B) percentage of small RNA species enriched in EVs. (C) Volcano plot shows no significant differences in miRNAs between EVs immunoprecipitated by anti‐CD81 antibodies from WJ‐MSC‐CD81 Snorkel‐tag and wildtype. (D) Volcano plot identifying single miRNA differentially expressed in Snorkel‐tag enriched EVs immunoprecipitated by anti‐HA and anti‐CD81 antibodies.
