# Supplementary figures and images for: Snorkel‐tag based affinity chromatography for recombinant extracellular vesicle purification
Source: J Extracell Vesicles. 2024 Oct 14;13(10):e12523. doi: 10.1002/jev2.12523 (PMC11472238; doi:10.1002/jev2.12523)

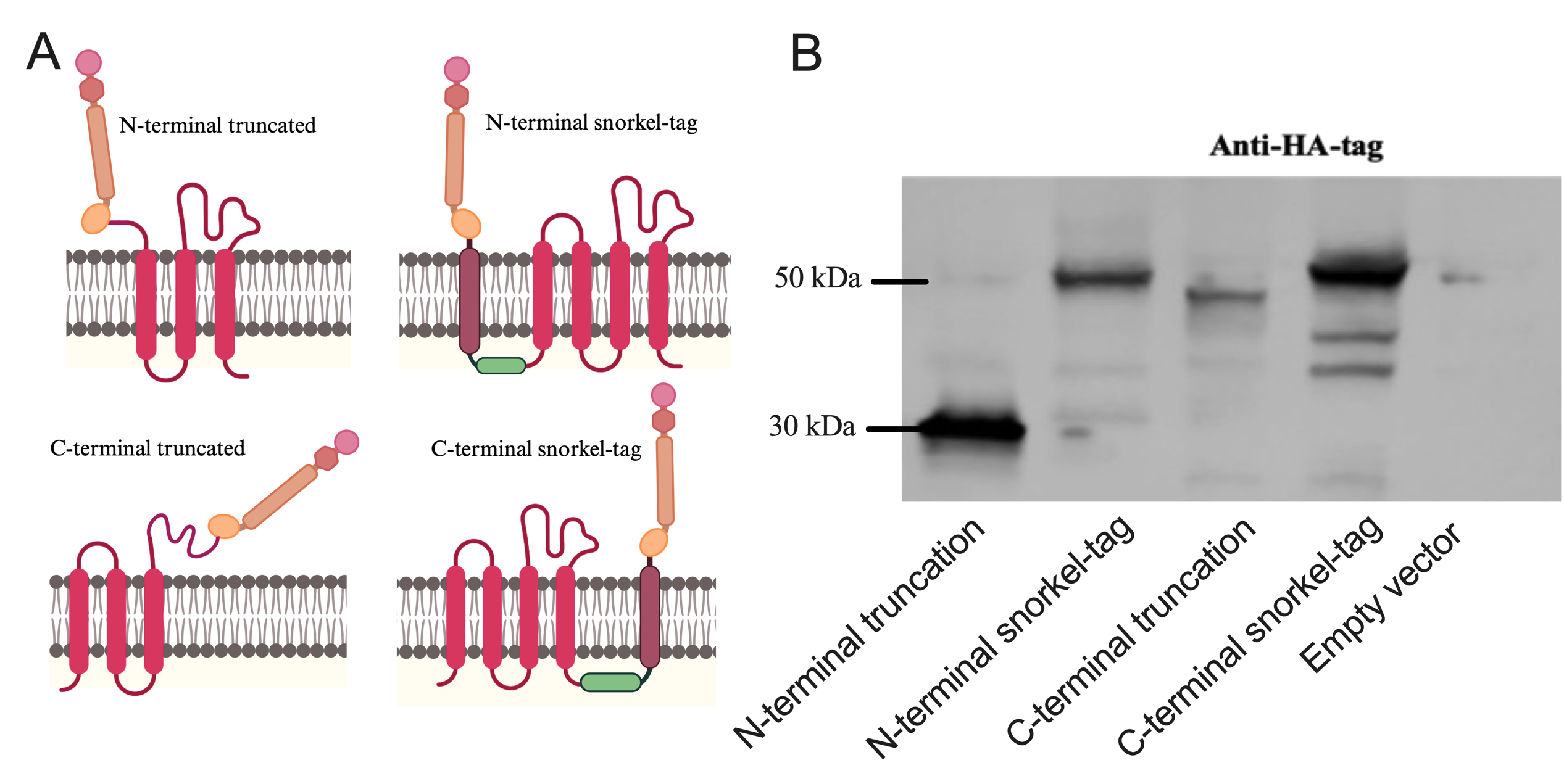

Supplement: Supplementary file 1 — Supplementary figure 1. (A) Schematic representation of full length CD81 genetically fused with Snorkel‐tag at C‐termini and CD81 truncated versions devoid of either transmembrane domains 1 or 4 with Snorkel‐tag fused to SEL (small extracellular loop) or LEL respectively. (B) Western blot of anti‐HA tag for all four fusion proteins transiently expressed in HeLa cells. Western blot results reveal N‐terminal truncated version of CD81 snorkel‐tag did not express full length protein. Created with BioRender.com. [file JEV2-13-e12523-s005.tiff]

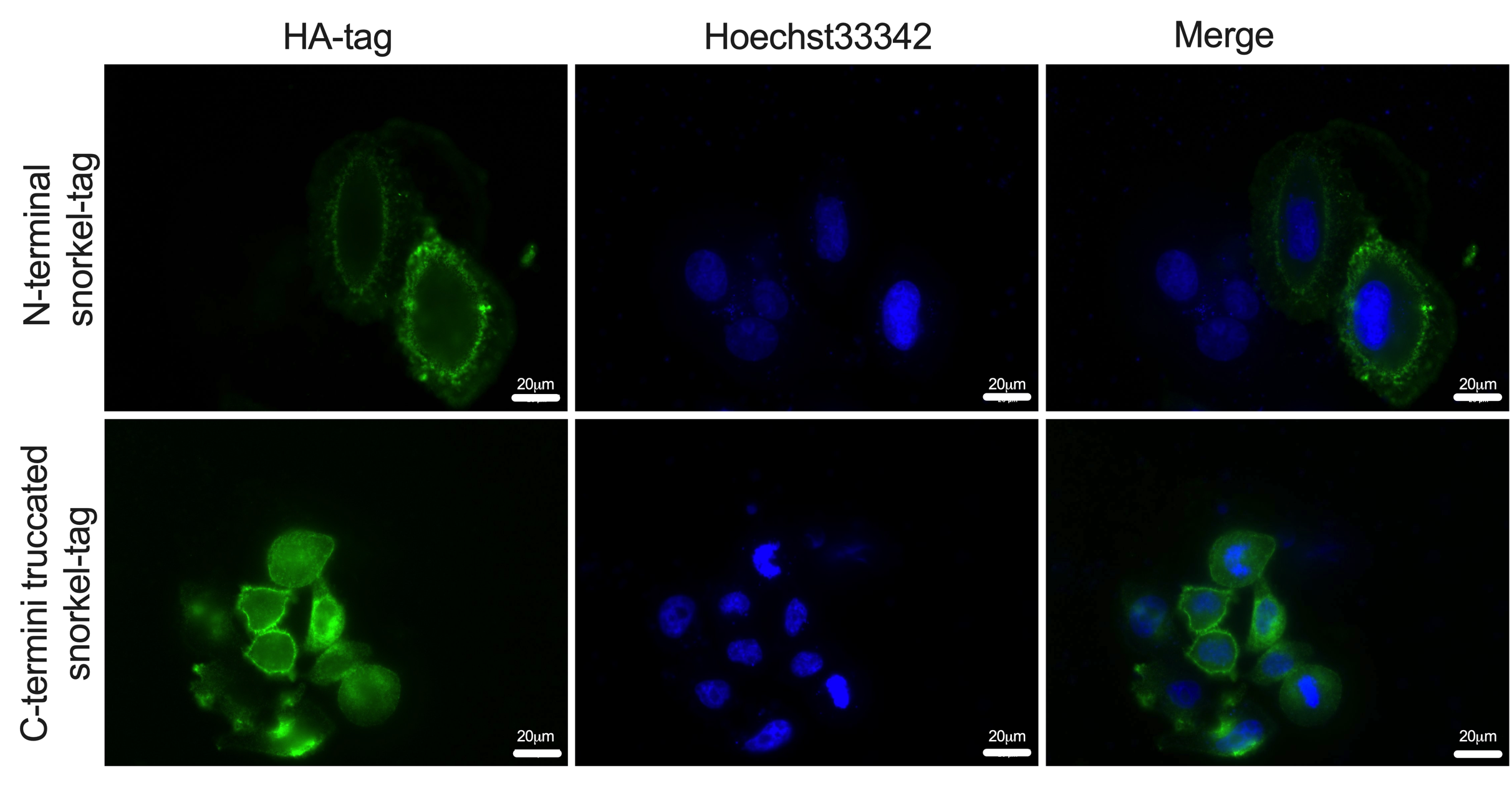

Supplement: Supplementary file 2 — Supplementary figure 2. Fluorescent images of fixed HeLa cells expressing CD81 with N‐terminal Snorkel‐tag and C‐terminal truncated Snorkel‐tag stained with anti‐HA tag antibody and Alexafluor‐488 anti‐rabbit secondary antibody. [file JEV2-13-e12523-s002.tiff]

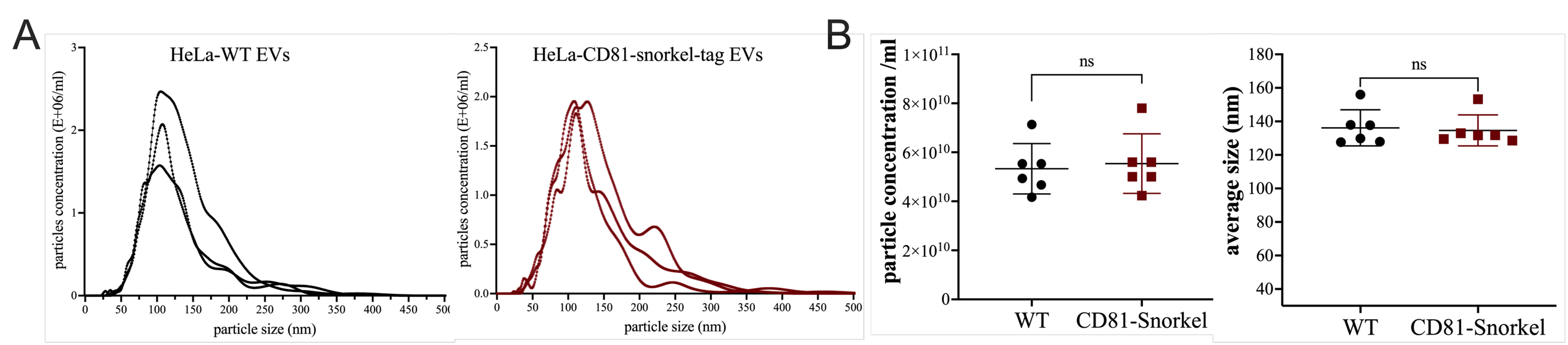

Supplement: Supplementary file 3 — Supplementary figure 3. (A) Representative particle size and concentration for EVs derived from HeLa‐WT and HeLa‐CD81‐Snorkel‐tag cell lines (n = 3). (B) Particle concentrations and size of ultrafiltrated particles from 75 mL conditioned media from 6 individual experiments. Unpaired t‐test was applied on raw values; nsP > 0.05. [file JEV2-13-e12523-s007.tiff]

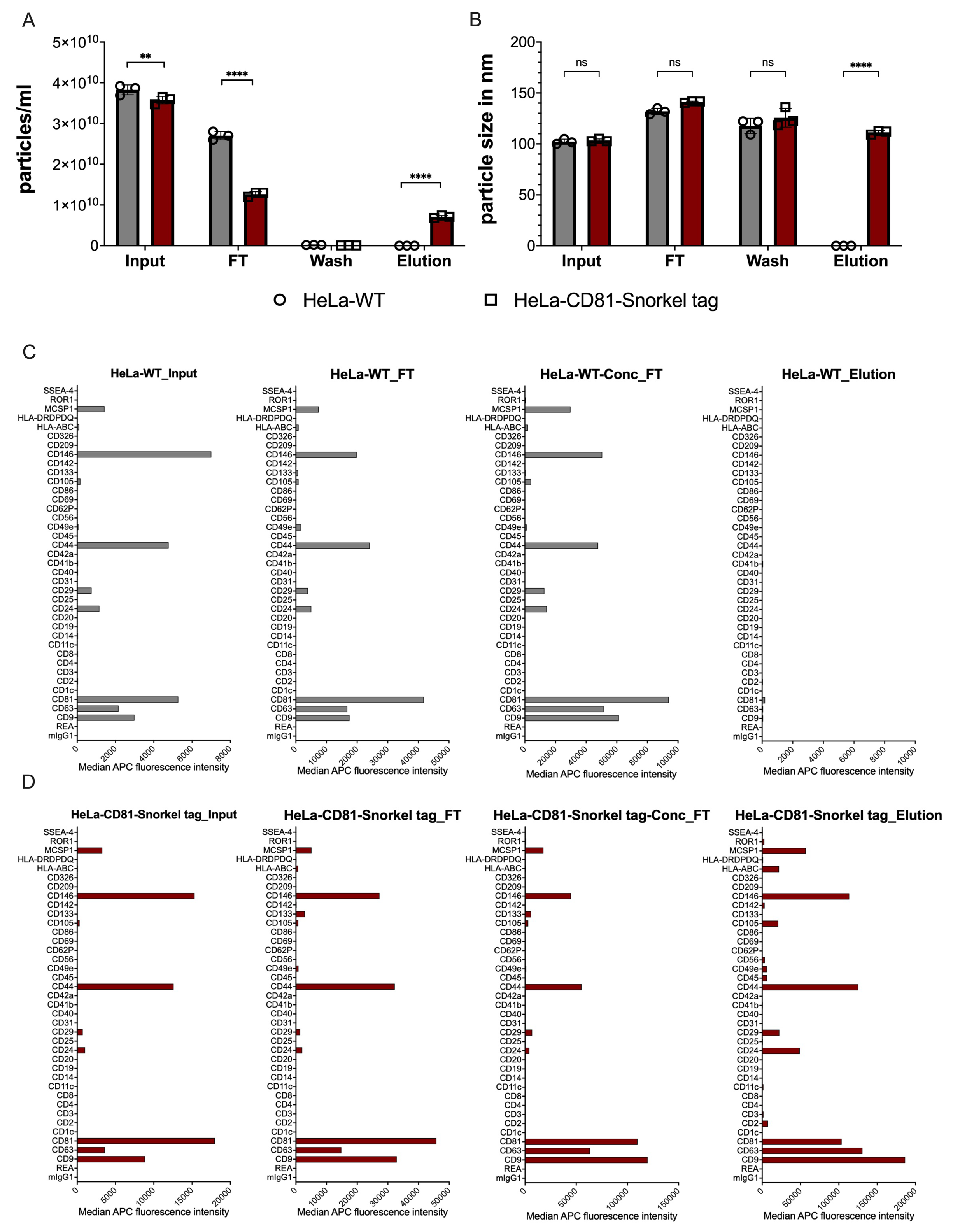

Supplement: Supplementary file 4 — Supplementary figure 4. Purification of Snorkel‐tag harbouring EVs from pre‐cleaned supernatants by StEVAC. NTA counts and particle diameter of purified EVs from supernatants (A) and (B). Multiplex bead‐based assay results for input, flow through, concentrated flow through and elution of EVs from HeLa‐WT (C) and Hela‐CD81‐Snorkel‐tag (D). 1‐way ANOVA was applied on raw values; nsP > 0.05, **p < 0.01, ***p < 0.001. [file JEV2-13-e12523-s003.tiff]

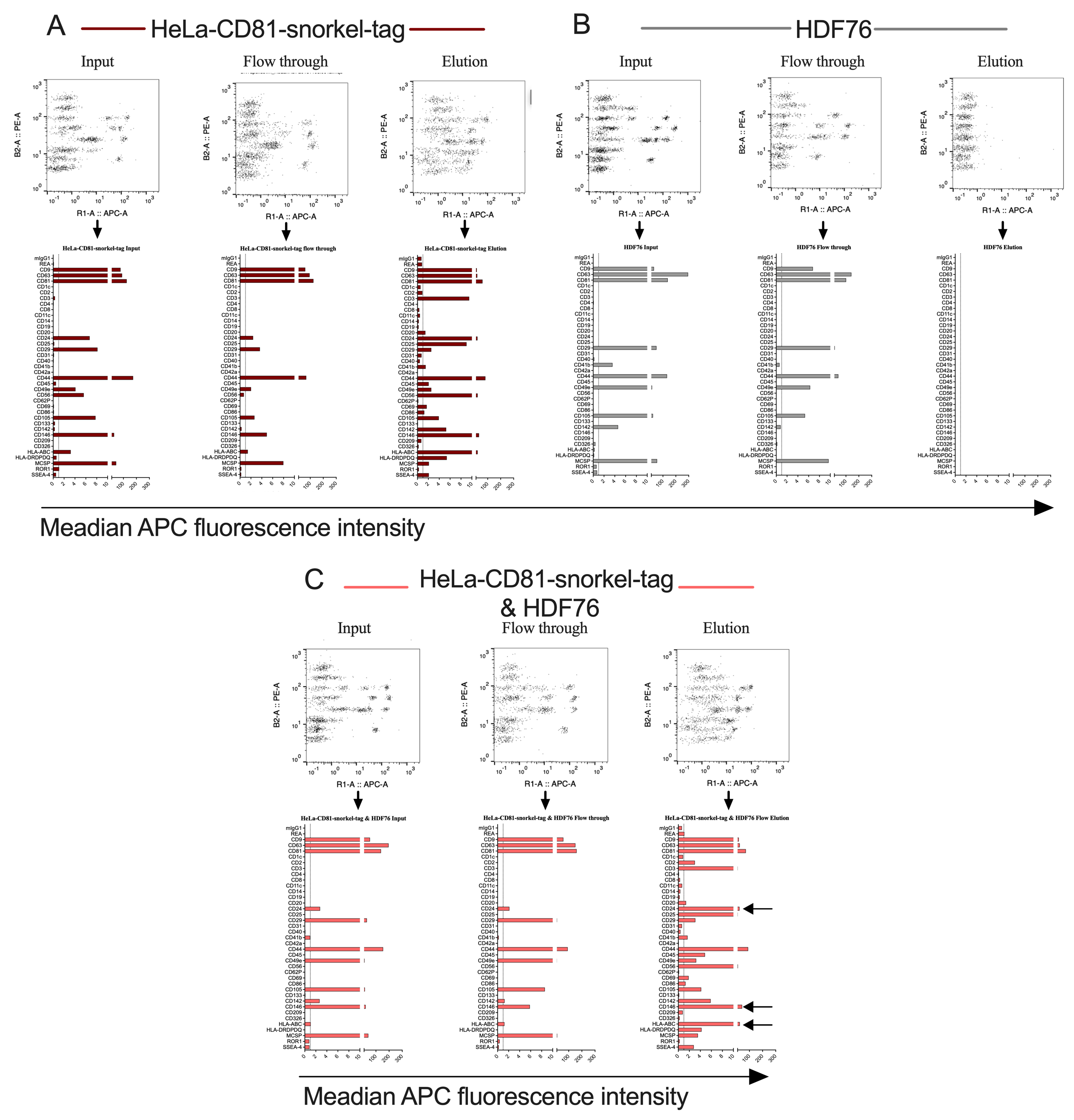

Supplement: Supplementary file 5 — Supplementary figure 5. Confirming StEVAC to purify EVs carrying Snorkel‐tag from mixed population of EVs. Multiplex bead‐based assay results for input, flow through and elution of EVs from HeLa‐CD81‐Snorkel‐tag (A); HDF76 (B) and HeLa‐ CD81‐Snorkel‐tag mixed with HDF76 (C). [file JEV2-13-e12523-s006.tiff]

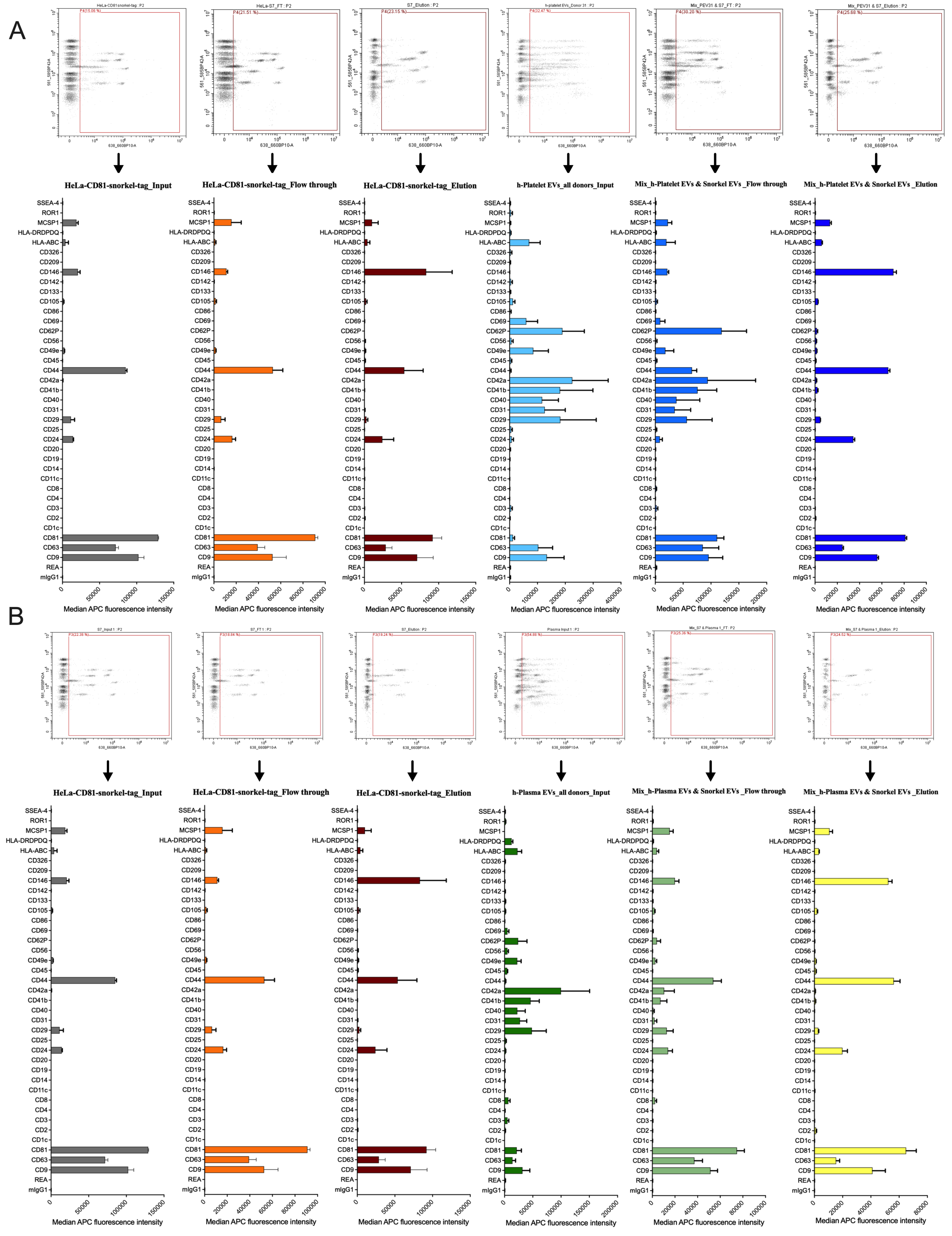

Supplement: Supplementary file 6 — Supplementary figure 6. MACSPlex 37 EV surface protein panel for HeLa‐CD81‐Snorkel‐tag and in mixtures with human platelet (A) and with human plasma (B); inputs, flowthroughs and elutes probed by anti‐pan tetraspanin APC antibodies (n = 3). [file JEV2-13-e12523-s001.tiff]

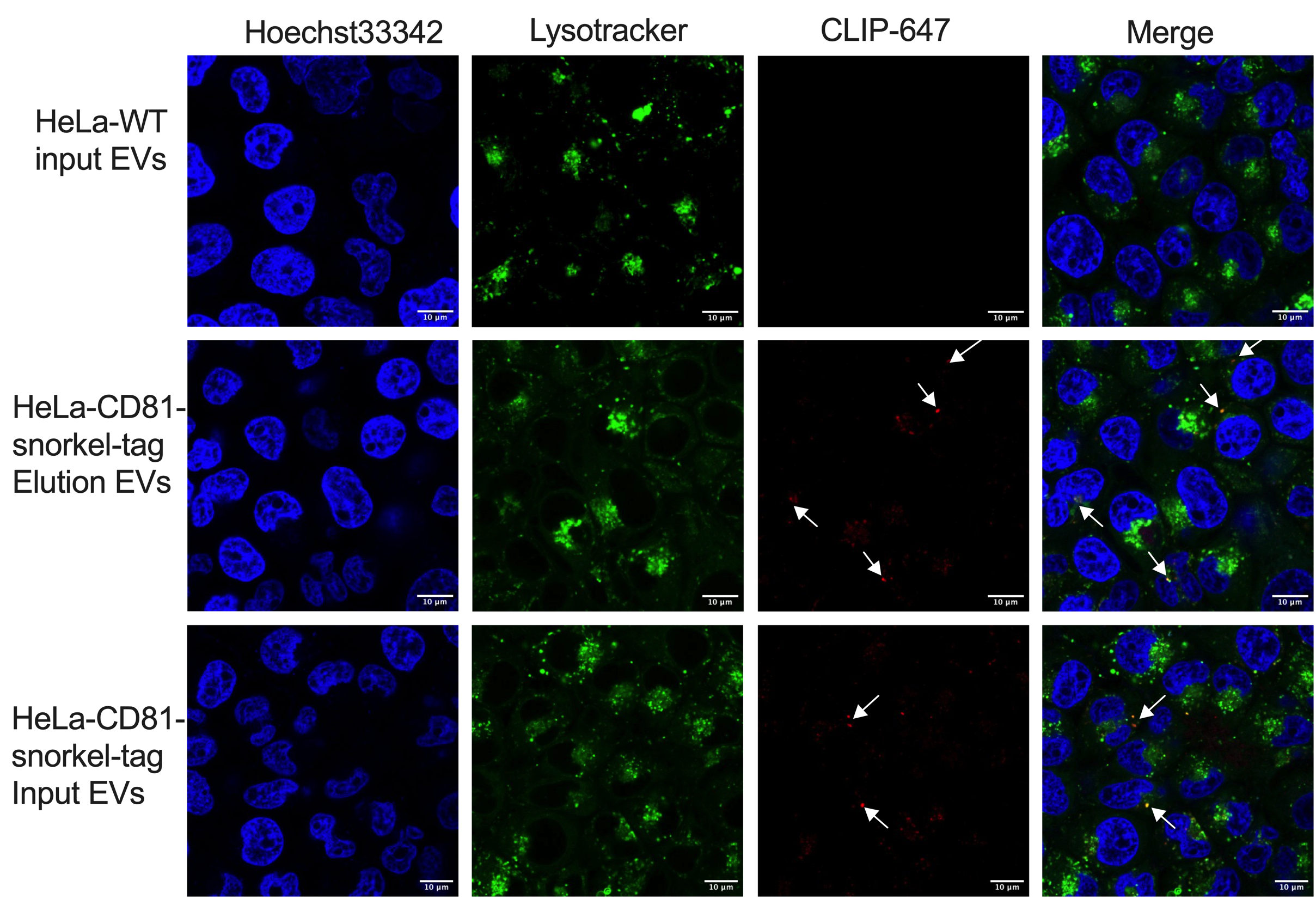

Supplement: Supplementary file 7 — Supplementary figure 7. Uptake of StEVAC purified EVs in Huh‐7 recipient cells. Representative Confocal images of StEVAC purified EVs labelled with CLIP‐647 uptake in Huh‐7 cells from HeLa‐WT, HeLa‐CD81‐Snorkel‐tag, as a positive control HeLa‐CD81‐Snorkel‐tag unpurified EVs in red. Counter staining with LysoTracker in green. [file JEV2-13-e12523-s008.tiff]

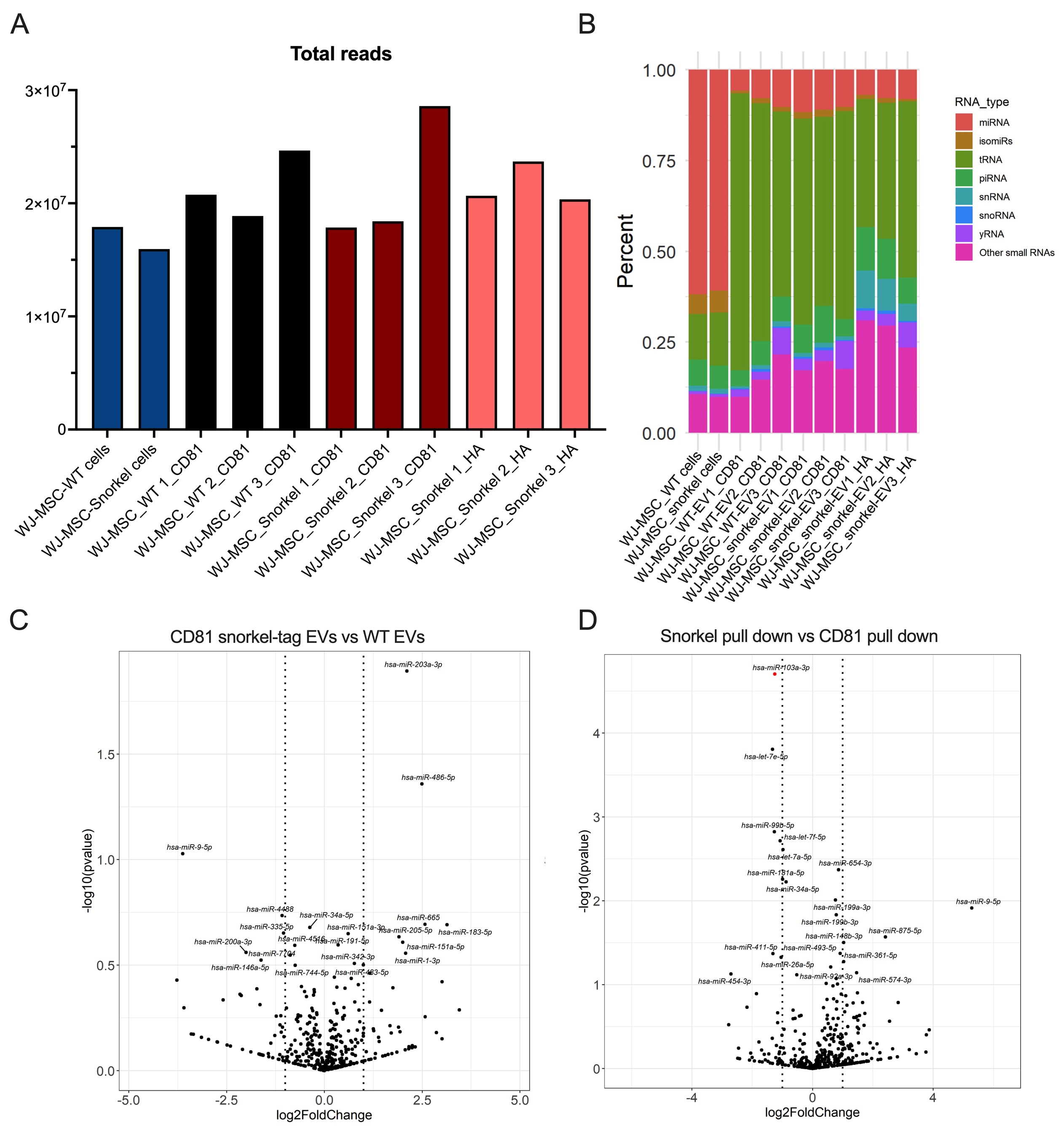

Supplement: Supplementary file 8 — Supplementary figure 8. EV cargo characterization. (A) total reads of small RNA sequencing from WJ‐MSC WT and Snorkel‐tag EVs pull down from Snorkel‐tag and CD81. (B) percentage of small RNA species enriched in EVs. (C) Volcano plot shows no significant differences in miRNAs between EVs immunoprecipitated by anti‐CD81 antibodies from WJ‐MSC‐CD81 Snorkel‐tag and wildtype. (D) Volcano plot identifying single miRNA differentially expressed in Snorkel‐tag enriched EVs immunoprecipitated by anti‐HA and anti‐CD81 antibodies. [file JEV2-13-e12523-s004.tiff]
